# Functional impact of cytochrome P450 3A (CYP3A) missense variants in cattle

**DOI:** 10.1038/s41598-019-56271-8

**Published:** 2019-12-23

**Authors:** Mery Giantin, Minna Rahnasto-Rilla, Roberta Tolosi, Lorena Lucatello, Marianna Pauletto, Giorgia Guerra, Francesca Pezzato, Rosa M. Lopparelli, Roberta Merlanti, Paolo Carnier, Francesca Capolongo, Paavo Honkakoski, Mauro Dacasto

**Affiliations:** 1 Department of Comparative Biomedicine and Food Science, Division of Pharmacology and Toxicology, University of Padua, Padua, Italy; 2 Faculty of Health Sciences, School of Pharmacy, University of Eastern Finland and Biocenter Kuopio, Kuopio, Finland; 3 Division of Pharmacotherapy and Experimental Therapeutics, Eshelman School of Pharmacy, University of North Carolina at Chapel Hill, Chapel Hill, NC USA

**Keywords:** Targeted resequencing, Expression systems

## Abstract

Cytochrome P450 3A is the most important CYP subfamily in humans, and CYP3A4/CYP3A5 genetic variants contribute to inter-individual variability in drug metabolism. However, no information is available for bovine CYP3A (bCYP3A). Here we described bCYP3A missense single nucleotide variants (SNVs) and evaluated their functional effects. *CYP3A28*, *CYP3A38* and *CYP3A48* missense SNVs were identified in 300 bulls of Piedmontese breed through targeted sequencing. Wild-type and mutant bCYP3A cDNAs were cloned and expressed in V79 cells. CYP3A-dependent oxidative metabolism of testosterone (TST) and nifedipine (NIF) was assessed by LC-MS/MS. Finally, SNVs functional impact on TST hydroxylation was measured *ex vivo* in liver microsomes from individually genotyped animals. Thirteen missense SNVs were identified and validated. Five variants showed differences in CYP3A catalytic activity: three *CYP3A28* SNVs reduced TST 6β-hydroxylation; one *CYP3A38* variant increased TST 16β-hydroxylation, while a *CYP3A48* SNV showed enhanced NIF oxidation. Individuals homozygous for *rs*384467435 SNV showed a reduced TST 6β-hydroxylation. Molecular modelling showed that most of SNVs were distal to CYP3A active site, suggesting indirect effects on the catalytic activity. Collectively, these findings demonstrate the importance of pharmacogenetics studies in veterinary species and suggest b*CYP3A* genotype variation might affect the fate of xenobiotics in food-producing species such as cattle.

## Introduction

Cytochrome P450 (CYP) enzymes are heme-containing proteins responsible for oxidation of drugs, environmental pollutants, food constituents as well as important endogenous compounds such as steroids and bile acids^1^. CYP3A is the most important CYP subfamily in humans, being involved in the phase I metabolism of more than 50% of therapeutic drugs^2^. It consists of four members, *i.e. CYP3A4*, *CYP3A5*, *CYP3A7* and *CYP3A43*, located in a cluster on chromosome 7. In particular, CYP3A4 and CYP3A5 share about 85% of sequence identity, highly similar substrate profiles and prominent expression in liver and intestine^3,4^. CYP3A7 is expressed in the fetal but not in the adult liver, while the expression of CYP3A43 is very low in human tissues^5^. The transcriptional regulation of human *CYP3As* in response to external signals has been characterized quite well: the nuclear receptors constitutive androstane receptor (CAR), pregnane X receptor (PXR) and peroxisome proliferator-activated receptor-alpha (PPAR-α) bind to their respective response elements in the *CYP3A* promoter region, modulating its expression (reviewed in^3^).

Increasing evidence shows that human *CYP3A* genetic variants may contribute to inter-individual variability in their metabolic activity, thus explaining adverse effects or unusual drug pharmacokinetics. A well-known example of inter-individual variability is represented by the intronic variant CYP3A4*22 (***rs*35599367**) that decreases CYP3A4 expression and activity^6^. Additionally, at least 24 single nucleotide variants (SNVs) in the *CYP3A4* coding region have been identified (https://www.pharmvar.org/gene/CYP3A4), albeit an effect on enzyme activity has been demonstrated only for few of them^2^. Complete loss-of-function mutations, such as CYP3A4*20, are very rare and detected in individuals with an unusual phenotype after exposure to CYP3A-metabolized drugs^7^. In contrast, the expression of CYP3A5 is highly variable among various ethnic groups, due to the common splicing variant CYP3A5*3 that inactivates the enzyme^8^.

The CYP3A subfamily has been well characterized in humans, rodents^3^ and pigs, a veterinary food-producing species considered as a reliable comparative model for human drug metabolism^9–13^. While studies on the variability of human drug-metabolising enzymes are focused on evaluation of clinical pharmacokinetics, drug responses and adverse effects, studies in animal species that are important sources of food products have further implications such as the risk assessment of harmful residues found in consumer food products^14–18^. In fact, food-producing animals are treated with drugs, they may substantially be exposed to agricultural pesticides or contaminants, and last but not least, their feed is supplemented with additives. The presence of all these substances and their metabolites in meat, milk or eggs represents a concern for the human health.

Cattle *(Bos taurus)* is one of the most important food-producing species worldwide. Bovine CYP3A (bCYP3A) enzymes are involved in the metabolism of a number of drugs widely used in farming such as the macrocyclic lactone moxidectin^19^, tiamulin and macrolide antibiotics^20^ as well as the ionophore monensin^21^. Moreover, bCYP3As are involved in the bioactivation of important natural toxins like aflatoxins and ergot alkaloids^22,23^. Three main b*CYP3A* genes have been identified, and a new nomenclature, mirroring the true evolutionary relationships among bCYP isoforms has been proposed: *CYP3A28* (orthologue of human *CYP3A4*), *CYP3A38* (orthologue of human *CYP3A5*) and *CYP3A48* (corresponding to “b*CYP3A4* nifedipine oxidase”)^24^. A fourth gene, annotated as *CYP3A24* within the b*CYP3A* cluster in chromosome 25, refers to a potential pseudogene^25^. The absolute quantification of liver mRNAs showed that CYP3A38 was the most abundantly expressed CYP3A isoform in bovine liver, followed by CYP3A48. Conversely, CYP3A28 (corresponding to the abundant human CYP3A4) was expressed at levels <1% in different cattle breeds^25^. Likewise to humans, physiological factors such as age, gender and breed have been shown to affect bCYP3A expression and/or activity^25–30^. Furthermore, a modulation of bCYP3A expression and catalytic activity after exposure to xenobiotics is well documented^25,31–34^; finally, there is recent evidence about the role of CAR and PXR in b*CYP3A* regulation^35,36^.

In contrast, scant information is available about the genetic variants affecting bCYP3A expression and activity. The three available studies investigated the effects of bCYP3A genetic variants on productive traits^23,37,38^. Therefore, our study aims to fill this scientific gap of knowledge by identifying missense mutations that could modify bCYP3A activity, with potential consequences on drug kinetics, therapeutic or adverse effects as well as on the levels of harmful residues in foodstuff. To this purpose, a pure Piedmontese cattle breed with precise individual pedigree information was selected. First, mutations within the b*CYP3A* gene cluster were identified through next-generation sequencing and then, individually validated by genotyping assays. Subsequently, the functional impact of the identified variants was evaluated by heterologous expression of bCYP3As and *in vitro* marker substrate metabolism. Moreover, *in silico* molecular modelling of wild-type (WT) and mutated (MUT) bCYP3As was performed. Finally, testosterone (TST) hydroxylation was determined in liver microsomes isolated from genotyped Piedmontese bulls.

## Results

### Sequence read and alignment statistics

More than 280 million high-quality reads were obtained. The total number of reads mapping against the bovine genome and used for the variant discovery was 150,616,772. The mean coverage measured in the b*CYP3A* cluster was 105X (maximum 254X and minimum 33X coverage). Alignment files (.bam) with reads mapped in the b*CYP3A* cluster have been deposited in the Sequence Read Archive (SRA) with the accession numbers **SRR7353738** - **SRR7353753**.

### Variant identification in the bCYP3A gene cluster

Data analysis identified 1,717 SNVs in the b*CYP3A* cluster that were distributed among exonic (25), intronic (500), intergenic (1,098), downstream (57), upstream (23) and undefined (12) regions and splicing sites (2), as well as 126 indels (43, 2 and 81 in the intronic, downstream and intergenic regions, respectively). Because the UMD 3.1 genome assembly originates from a single Hereford breed animal, annotation of the SNVs was verified in the Ensembl Genome Browser and NCBI databases to detect SNVs putatively specific for the Piedmontese breed; 204 novel SNVs (12% of total) were identified.

Since we focused on the missense variants potentially affecting the enzyme activity, only such coding variants (n = 13, Table 1) were functionally evaluated. Out of these, ten SNVs were already present in the databases, while the remaining three (CYP3A38_11A Glu374Asp, CYP3A38_11B Phe376Leu and CYP3A48_7 Glu311Lys) were identified for the first time.

**Table 1:** Missense variants identified in b*CYP3As*.

GENE	SNV missense mutation	REF ENSEMBL GENOME BROWSER location	NCBI ID
*CYP3A28*	CYP3A28_7 Gly197Ser	ENSBTAT00000063483.1:exon7:c.G589A:p.G197S	***rs*384467435**
	CYP3A28_10 Ala289Val	ENSBTAT00000063483.1:exon10:c.C866T:p.A289V	***rs*433125080**
	CYP3A28_11 Ile388Val	ENSBTAT00000063483.1:exon11:c.A1162G:p.I388V	***rs*454167819**
*CYP3A38*	CYP3A38_8 Val253Ile	ENSBTAT00000007170.5:exon8:c.G757A:p.V253I	***rs*440751676**
	CYP3A38_11A Glu374Asp	ENSBTAT00000007170.5:exon11:c.G1122T:p.E374D	N.A.
	CYP3A38_11B Phe376Leu	ENSBTAT00000007170.5:exon11:c.T1126C:p.F376L	N.A.
*CYP3A48*	CYP3A48_5 Asn180Thr	ENSBTAT00000016177.5:exon5:c.A539C:p.N180T	***rs*384714392**
	CYP3A48_6A His220Asp	ENSBTAT00000016177.5:exon6:c.C658G:p.H220D	***rs*381315842**
	CYP3A48_6B Val225Leu	ENSBTAT00000016177.5:exon6:c.G673C:p.V225L	***rs*110013281**
	CYP3A48_7 Glu311Lys	ENSBTAT00000016177.5:exon7:c.G931A:p.E311K	N.A.
	CYP3A48_8A Asn316Lys	ENSBTAT00000016177.5:exon8:c.T948G:p.N316K	***rs*384081812**
	CYP3A48_8B Val351Ile	ENSBTAT00000016177.5:exon8:c.G1051A:p.V351I	***rs*137124349**
	CYP3A48_9 Gly391Asp	ENSBTAT00000016177.5:exon9:c.G1172A:p.G391D	***rs*384367918**

N.A. = not available.

### Validation of missense SNVs and genotyping of individuals

SNV-specific genotyping assays allowed validation of the thirteen missense variants that were identified by re-sequencing of the b*CYP3A* cluster. Almost all SNVs were confirmed to be present at least as a heterozygote in the population tested, confirming the sequencing data. The only exception was the variant CYP3A28_10 Ala289Val that was indeed flagged as a low-quality SNV after filtering, and for which the genotyping assay was not successful in detecting the mutation. Two SNVs identified with a low confidence in sequencing (CYP3A38_8 Val253Ile and CYP3A48_7 Glu311Lys) were properly genotyped but detected only as heterozygotes.

Table 2 shows genotypes frequencies, Chi square test and MUT allele’s frequency detected in the tested population. The homozygous MUT/MUT genotype was observed only for seven SNVs with a frequency higher than 1%. Variant alleles frequencies that were calculated based on Hardy-Weinberg Equilibrium (HWE) were always above 0.1. Interestingly, five of them (CYP3A28_7 Gly197Ser, CYP3A48_5 Asn180Thr, CYP3A48_6A His220Asp, CYP3A48_6B Val225Leu, CYP3A48_8A Asn316Lys) were not consistent with the HWE (see Table 2).

**Table 2:** Genotypes and mutant alleles frequency of b*CYP3A* SNVs in the Piedmontese cattle cohort.

SNV ID	Genotypes Frequency (WT/WT – WT/MUT – MUT/MUT)	Chi-square test **p-value < 0.01 ***p-value < *0.001*	MUT allele’s frequency
CYP3A28_7 Gly197Ser		8.95**	0.12
CYP3A28_10 Ala289Val		Not applicable	
CYP3A28_11 Ile388Val		Not applicable	
CYP3A38_8 Val253Ile		Not applicable	
CYP3A38_11A Glu374Asp		Not applicable	
CYP3A38_11B Phe376Leu		Not applicable	
CYP3A48_5 Asn180Thr		Not applicable	
CYP3A48_6A His220Asp		35.11***	0.10
CYP3A48_6B Val225Leu		12.42***	0.47
CYP3A48_7 Glu311Lys		Not applicable	
CYP3A48_8A Asn316Lys		7.84**	0.21
CYP3A48_8B Val351Ile		1.57	0.16
CYP3A48_9 Gly391Asp		0.76	0.27

Hardy-Weinberg Equilibrium (HWE) was assessed by means of Chi-square test. Asterisks highlight significant deviations from HWE. WT/WT, WT/MUT and MUT/MUT genotypes frequency are represented by white, light and darker gray bars, respectively.

The remaining six SNVs (except the CYP3A28_10 Ala289Val described above) showed the variant nucleotide only in heterozygote genotype, with frequencies in the range 0.3–40.3%. Noteworthy, the three novel SNVs in Piedmontese cattle breed showed the lowest frequencies, as they were represented only in one (CYP3A38_11A Glu374Asp) or seven (CYP3A38_11B Phe376Leu and CYP3A48_7 Glu311Lys) individuals out of 300 (0.3 and 2.3%, respectively).

### Catalytic activity of wild-type CYP3A isoforms in V79 recombinant cells

After confirmation by Sanger sequencing, full-length wild-type b*CYP3A*s and bovine cytochrome P450 oxidoreductase (b*POR*) clones were heterologously expressed in V79 cells, optimized for CYP:POR ratio (10:1), substrate concentration and length of incubation. Transfection and correct translation of bCYP3As was verified by observing properly sized protein by immunoblotting and detectable enzyme activity.

Among the three wild-type CYP3A isoforms, only CYP3A28 and CYP3A38 were able to hydroxylate TST (Supplementary Fig. S1). The main metabolite produced by CYP3A28 was 6β-hydroxytestosterone (6β-OH TST) (Supplementary Fig. S1c). Comparison of the LC-MS/MS chromatograms CYP3A28-transfected cells showed a significant increase (~16-fold over native cells) in 6β-OH TST production. Traces of this metabolite in control cells were attributed to an impurity of the analytical standard and not to the basal activity of V79 cells, because this metabolite was detected also in the spiked samples used for the standard curve.

However, CYP3A38 produced both hydroxylated TST metabolites, with a preference to the 16β-hydroxytestosterone (16β-OH TST) (Supplementary Fig. S1d). While the amount of 6β-OH TST formed by CYP3A38 was low (only ~4.5-fold above control cells) and well below that by CYP3A28, 16β-OH TST was completely absent in the medium of native V79 cells and appeared only after CYP3A38 transfection.

CYP3A48 produced very small amounts of 6β-OH and 16β-OH TST and only traces (below the limit of quantitation, LOQ) of other, presumably hydroxylated TST metabolites (likely 2β-OH and 12β-OH TST; Supplementary Fig. S1e). Hence, another CYP3A marker substrate NIF was selected for evaluating CYP3A48 activity. Preliminary investigations showed that NIF was clearly oxidized by CYP3A28 and CYP3A38; nevertheless, biotransformation by CYP3A48 was slow. CYP3A48 was able to slightly increase NIF oxidation (Supplementary Fig. S2, grey peak). The other peaks, present in both native (Supplementary Fig. S2b) and CYP3A48-transfected cells (Supplementary Fig. S2c), were suggestive of further metabolites or products of NIF degradation, possibly due to its sensitivity to light and temperature.

### Impact of missense SNVs on the CYP3A catalytic activity

Wild-type *CYP3A28*, *CYP3A38* and *CYP3A48* clones were subjected to site-directed mutagenesis to incorporate each of the 13 missense SNVs. The *CYP3A* variants were expressed in V79 cells and their expression was verified by immunoblotting. All CYP3A28, CYP3A38 and CYP3A48 mutant proteins were efficiently translated in V79 cells and expressed at comparable levels to the appropriate WT isoform.

**Figure 1:**
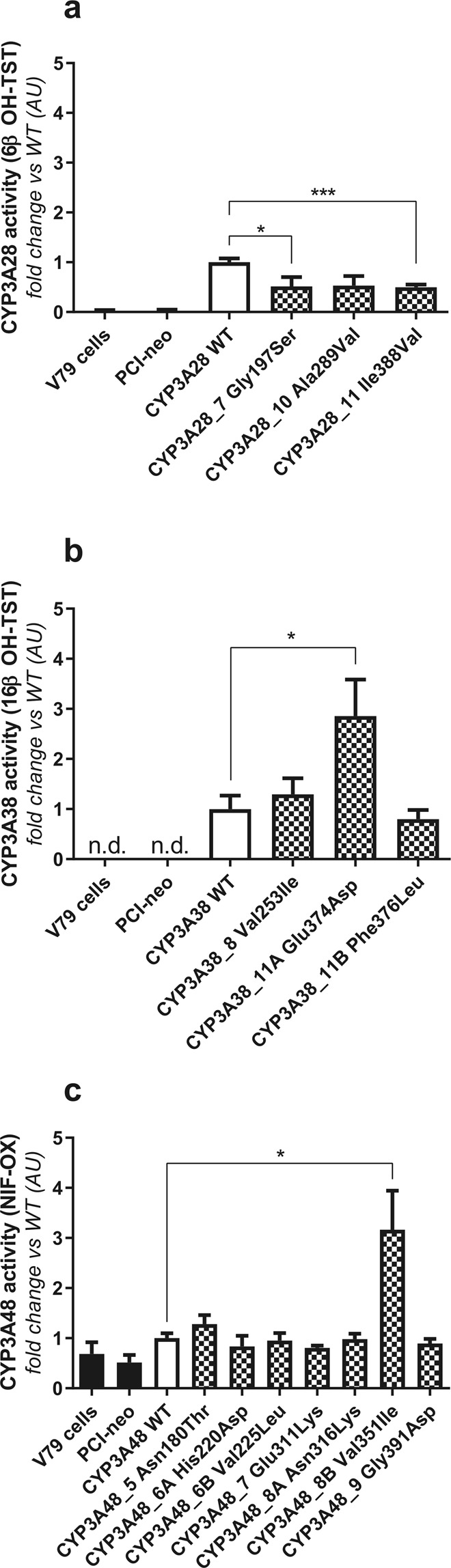
Catalytic activity of V79 cells expressing wild-type and mutant (**a**) CYP3A28, (**b**) CYP3A38 and (**c**) CYP3A48. After incubation of transfected V79 cells with 25 µM TST or 5 µM NIF for 3 or 1.5 hours, the medium was subjected to LC-MS/MS analysis as reported in the Material and Methods. The catalytic activity of wild-type and mutant CYP3A28, CYP3A38 and CYP3A48 proteins was calculated dividing the amount of metabolite formed by the incubation time and the total protein content (nmoles min^−1^ mg^−1^ protein). Variant CYP3A specific activities were normalized to that of the wild-type CYP3A isoform (set at 1.00). Four independent experiments were performed. Unpaired T-test was used for statistical analysis (*p < 0.05; ***p < 0.001). AU: Arbitrary Units.

The catalytic activities of variant CYP3As proteins were subsequently evaluated by measurement of TST or NIF metabolism by recombinant cells (Fig. 1). TST 6β-hydroxylation was significantly reduced (~50%) in V79 cells transfected with CYP3A28_7 Gly197Ser (*p* < 0.05) and CYP3A28_11 Ile388Val (*p* < 0.01) clones when compared to cells transfected with the wild-type CYP3A28 (Fig. 1a). The same trend was observed in cells transfected with CYP3A28_10 Ala289Val, although the reduction in TST 6β-hydroxylation was not significant (*p* = 0.0522). Conversely, none of CYP3A38 SNVs affected 6β-OH TST production (Supplementary Fig. S3). For TST 16β-hydroxylation, a significant increase (~3-fold, *p* < 0.05) was obtained only for CYP3A38_11A Glu374Asp (Fig. 1b).

Finally, NIF was used for evaluating the functional impact of CYP3A48 SNVs (Fig. 1c). NIF oxidation was rather similar among CYP3A48 variants and close to the native V79 or cells transfected with the empty PCI-neo vector, confirming the preliminary results for wild-type CYP3A48. The only exception was CYP3A48_8B Val351Ile, whose activity was the highest among the seven CYP3A48 SNVs tested (~3-fold increase over wild-type CYP3A48, *p* < 0.05).

### Impact of CYP3A missense SNVs on the bovine liver microsomal TST hydroxylation

To investigate if the identified missense SNVs could affect the metabolism *ex vivo*, TST hydroxylation was evaluated in liver microsomes isolated from 300 genotyped Piedmontese bulls.

**Figure 2:**
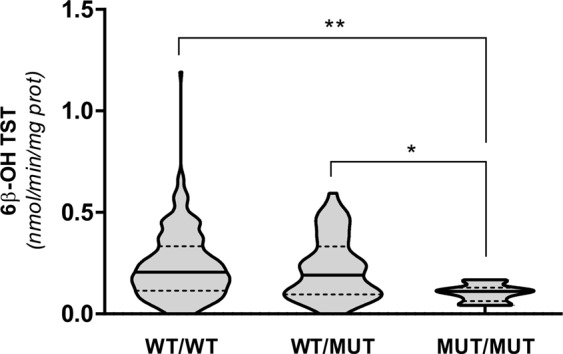
6β-OH TST production in liver microsomes isolated from Piedmontese bulls genotyped for CYP3A28_7 Gly197Ser. The catalytic activity was calculated in nmoles min^−1^ mg^−1^ protein. ANOVA followed by Tukey’s post-test was used for statistical analysis (*p < 0.05; **p < 0.01). Black and dotted lines represent median values and quartiles, respectively.

Among the five SNVs impacting bCYP3A catalytic activity in recombinant cells, only CYP3A28_7 Gly197Ser was shown to modulate the hepatic microsomal activity. Liver microsomes isolated from animals homozygous for this variant allele produced significantly lower amounts of 6β-OH TST when compared to WT/WT and WT/MUT bulls (*p* < 0.01 and *p* < 0.05, respectively, Fig. 2), but similar amounts of 16β-OH TST (Supplementary Table S1). To corroborate this results, CYP3A immunoblotting analyses were performed in microsomal proteins isolated from a representative number (n = 30) of genotyped Piedmontese cattle (Supplementary Fig. S4); no differences were observed among WT/WT, WT/MUT and MUT/MUT groups. Conversely, CYP3A28_11 Ile388Val and CYP3A48_8B Val351Ile did not appear to affect TST metabolism in Piedmontese cattle (Supplementary Table S1). No comparisons could be made for CYP3A28_10 Ala289Val and CYP3A38_11A Glu374Asp, as the variant alleles were either not detected or present in one heterozygous animal only.

For the remaining eight SNVs, no statistically significant differences in the catalytic activity were observed among different genotypes in either *ex vivo* or *in vitro* studies (Supplementary Table S1).

### Molecular modelling of TST and NIF docked into wild-type and mutant CYP3As

**Figure 3:**
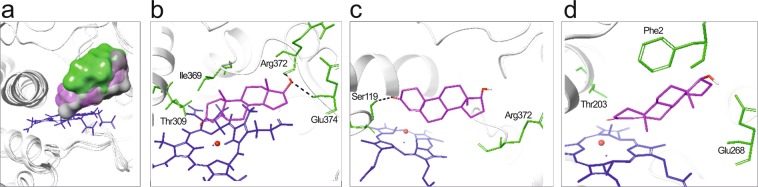
The docking of TST to bCYP3A enzymes. (**a**) The location of the substrate pocket in wild-type CYP3A28 (*grey cloud*), CYP3A38 (*green cloud*) and CYP3A48 (*magenta cloud*) isoforms. (**b–d**) Close-up view of interactions of TST (*magenta*) in the substrate pockets of (**b**) CYP3A28, (**c**) CYP3A38 and (**d**) CYP3A48. Only the most important pocket residues (*green*) are shown for clarity. Black dashes indicate hydrogen bonding, heme is in *blue* and CYP backbone in *grey* ribbon.

All bCYP3A protein models showed typical features of CYP structures. Homologies between the human CYP3A4 and the wild-type CYP3A28, CYP3A38 and CYP3A48 isoforms were 72%, 69% and 59%, respectively. The docking poses of CYP3A models (Fig. 3) revealed that TST occupied a large binding pocket close to the heme group and usually oriented its A ring towards the heme. A minority of the poses for CYP3A38 models indicated an opposite orientation where the steroid D ring was directed towards the heme (Supplementary Fig. S5).

However, TST was closer to the heme iron within the CYP3A28 binding pocket (distance between the carbon-6 and Fe atoms 6.2 Å), as compared with CYP3A38 (8.2 Å) and CYP3A48 (6.9 Å) (Fig. 3a). A closer investigation showed that TST located near hydrophobic residues such as Ile369, Ala370 and Leu373. Here, the A ring of TST was oriented so that the typical site for CYP3A-mediated hydroxylation (carbon-6) was projected towards the heme iron. The majority of CYP3A28 docking poses displayed hydrogen bonding between TST and Arg372 (Fig. 3b and Supplementary Fig. S6), while CYP3A38 docking indicated hydrogen bonding with Ser119 (Fig. 3c and Supplementary Fig. S7). In contrast, TST did not show any hydrogen bonding in the CYP3A48 substrate pocket (Fig. 3d). In addition, the CYP3A28 scoring poses suggest a better binding affinity for TST than those of CYP3A38 or CYP3A48. The ranking of scoring poses also matched the TST 6β-hydroxylation activities of these CYP3A enzymes (Table 3).

**Table 3:** Docking scores of TST and TST hydroxylation activities.

	Docking Score	Docking poses ratio^a^	6β-OH TST	16β-OH TST
Wild-type CYP3A28	−10.1	10	0.091	n.d.
CYP3A28_7 Gly197Ser	−9.1	n.d.	0.057	n.d.
CYP3A28_10 Ala289Val	−7.1	n.d.	0.056	n.d.
CYP3A28_11 Ile388Val	−9.7	n.d.	0.043	n.d.
Wild-type CYP3A38	−7.5	2.5	0.010	0.012
CYP3A38_8 Val253Ile	−10.3	5	0.014	0.016
CYP3A38_11A Glu374Asp	−7.4	2.5	0.009	0.031
CYP3A38_11B Phe376Leu	−8.4	10	0.012	0.010
Wild-type CYP3A48	−6.8	n.d.	traces	traces

^a^Approximate ratio of docking poses with carbon-6 to carbon-16 oriented towards heme.

Activities are expressed as nmoles min^−1^ mg^−1^ protein with blank reactions subtracted.

n.d.: not detectable.

The majority of CYP3A28, CYP3A38 and CYP3A48 SNVs here identified were located at the surface of the protein, thus far from the binding pocket of the enzyme (Supplementary Fig. S8). Only the residue in CYP3A38_11A Glu374Asp was less than 10 Å from the heme. The docking poses of TST were quite similar near the binding pocket residues Ile369, Ala370 and Leu373 and Arg105, Ser119 and Thr309 for wild-type and mutant CYP3A28 models (Supplementary Fig. S6). Also, the docking poses of TST near common Arg105, Ser119 and Arg372 residues were similar within the different CYP3A38 models (Supplementary Figs. S5 and S6). In a minority of poses, they also projected the steroid D ring of TST towards the heme (distance between the carbon-16 and Fe atoms were between 4–5 Å) supporting the appearance of TST 16β-hydroxylation for CYP3A38 enzymes (Supplementary Figs. S5 and S9). However, the docking scores between the wild-type and mutant CYP3A28 and CYP3A38 isoforms did not show a clear correlation with the catalytic activity (Table 3).

**Figure 4:**
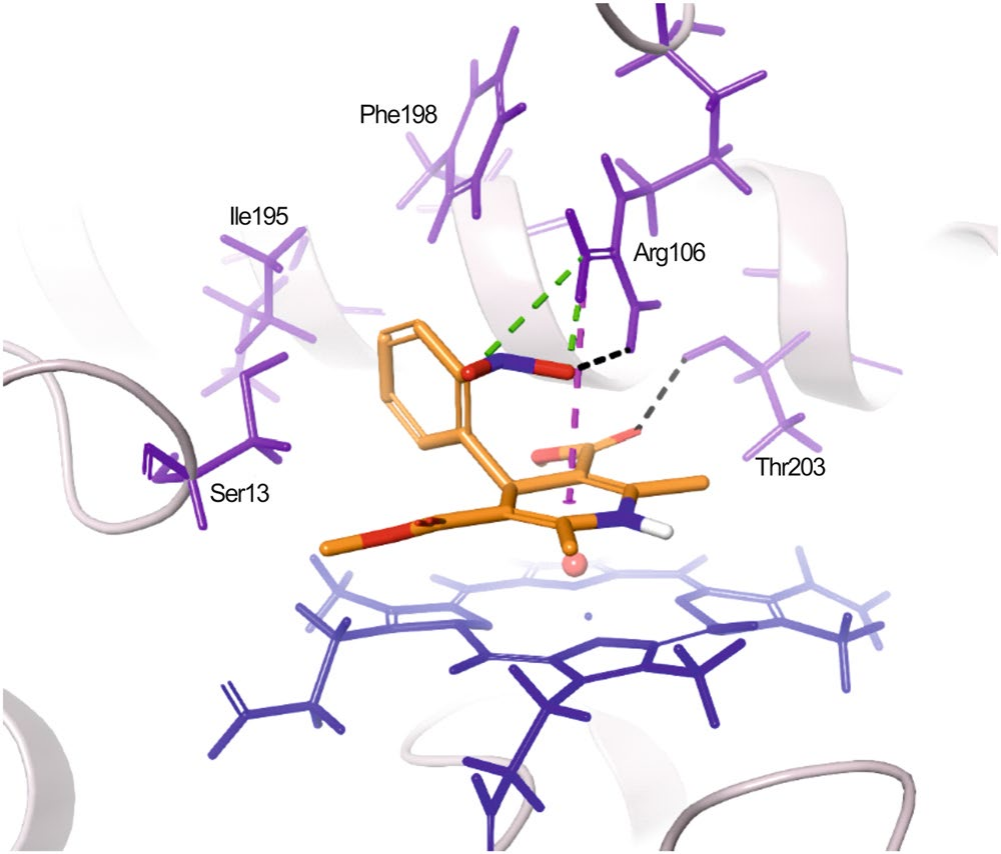
NIF-amino acid interactions in the CYP3A48 substrate pocket. Residues that line the substrate-binding pocket are highlighted by *purple*, the heme group is shown by *blue* and the CYP backbone by *grey* ribbon. *Black* dashes indicate hydrogen bonding, *green* dashes salt bridges and *magenta* dashes π-cation interactions.

NIF could be docked to the wild-type and mutant CYP3A48 substrate pocket. Overall, orientations of docking poses were quite similar for both wild-type and mutant models (Fig. 4 and Supplementary Fig. S9). They projected the NIF N1 atom towards the heme with the exception of CYP3A48_7 Glu311Lys mutant. Some docking poses showed π-cation and salt bridge interactions with Arg106. CYP3A48_5 Asn180Thr and CYP3A48_8B Val351Ile displayed two hydrogen bonds with Arg106 and Ser13 while the wild-type CYP3A48, CYP3A48_6B Val225Leu and CYP3A48_8B Val351Ile formed only one hydrogen bond with Thr203 or Ser13. The docking scores between wild-type and mutant CYP3A48 forms did not show a trend with the NIF N-oxidation activity.

## Discussion

CYP3A is the most important CYP subfamily in adult humans because of its prominent expression in liver and gut and its broad substrate specificity^3^. Compared to humans, bCYP3A seems of minor importance even though it is involved in the biotransformation of important veterinary drugs used in clinical practice (*e.g*., moxidectin, tiamulin, macrolide antibiotics and monensin^19–21^. It also contributes to the bioactivation of important natural toxins such as aflatoxins and ergot alkaloids^22,23^. Moreover, hepatic CYP3A expression and/or activity has been shown to be affected to a varying extent in veal and beef cattle by illicit growth promoting schedules^31,32,39^. Finally, bCYP3A seems to be involved in physiological mechanisms associating nutrition and reproductive function in dairy cows^40–42^.

Despite this, information on *CYP3A* genetic variability are quite limited, and usually referable to the effects of bCYP3A variants on productive traits. As an example, Brahman cattle are consistently less sensitive to ergot alkaloids associated with endophyte-infected tall fescue than Angus cattle^37^. Since CYP3A subfamily metabolizes ergot alkaloids, a relationship between a CYP3A variant and the sensitivity to ergot alkaloids was hypothesized; nevertheless, the functional impact of this variant was mostly associated with milk yield and composition and calving traits^23,37,38^. Apart from these studies, experiments characterizing bCYP3A mutations functionally or *in silico* have never before been performed. Therefore, we attempted to analyse missense mutations in the b*CYP3A* cluster and to unveil their effects on CYP3A-mediated metabolism. To reach this goal, a genetically selected Piedmontese breed was preferred, because it facilitated the identification of rare b*CYP3A* mutations that could be masked by heterozygous genotypes in cross-bred individuals.

The mean sequencing coverage was very high (average 105X) which allowed us to confirm location and correct orientation of *CYP3A28*, *CYP3A38* and *CYP3A48* genes in chromosome 25 (positions 37,005,334–37,319,692) and identify about 1,700 SNVs in our cohort of 300 bulls. These variants were largely distributed in intronic (63.9%) and intergenic regions (29%), but also within exonic and promoter regions as well as in splicing sites (7.1%). About 12% of SNVs were novel and not present in the public databases. This result might suggest that this relatively small number of novel SNVs are specific for the Piedmontese breed.

Here, we found a total of 13 missense SNVs (three in *CYP3A28*, three in *CYP3A38* and seven in *CYP3A48*). Three of these variants (CYP3A38_11A Glu374Asp, CYP3A38_11B Phe376Leu, CYP3A48_7 Glu311Lys) were identified for the first time in the bovine genome. Further genotyping analyses might resolve whether these mutations are specific to the Piedmontese breed or also present in different breeds. Notably, our investigations on the Limousine breed from the Veneto region in Italy have shown that the CYP3A38_11A Glu374Asp variant is missing in this breed (Tolosi *et al*., submitted).

The re-sequencing approach used in the present study enabled an accurate variant detection, because almost all SNVs were confirmed and validated by HybProbe genotyping and Sanger sequencing. The genotyping failed to detect only the CYP3A28_10 Ala289Val variant. Three different assays were tested but none could identify this variant allele. This might imply that (1) either the CYP3A28_10 Ala289Val is a very rare variant or (2) the genotyping assays designed for this DNA region are not sensitive and accurate enough. This variant is characterized by a long stretch of T nucleotides very close to the upstream intronic region, and this might affect probe binding.

Overall, the MUT alleles were detected as homozygotes with a frequency >1% for seven SNVs, while only heterozygotes were present for the remaining five SNVs. This might lead to two different interpretations: (1) the five SNVs are very rare in the Piedmontese cattle, and only a screening of a larger population will identify the MUT/MUT genotype, and (2) due to selective pressure (either natural or man-driven), the minor allele is disappearing.

Four out of the seven variants violated the HWE. Departures from HWE are possibly due to either strong genetic selection and mating systems imposed to the populations under selection (such as inbreeding) or a small sampling size^43,44^. Notably, three variants deviating from HWE showed loss of heterozygosity, which is often associated with inbreeding. Considering that the Piedmontese breed is the result of years of human selection for production traits, adaptation to different environments and demographic effects as domestication, migration and selection^45^, a change in the allelic balance is to some extent expected^43,46^.

No published data on the wild-type or mutant bCYP3A enzyme activity are available, so we expressed these proteins in the V79 cell line. These cells express POR and cytochrome b5 (necessary for CYP3A activity) endogenously but have low CYP levels. Therefore, V79 cells have often been used to study human and rat CYP enzymes^47–50^, a bovine CYP3A4-like isoform^51^ and equine isoforms CYP3A89, CYP3A94 and CYP3A95^52^. Despite endogenous cytochrome P450 oxidoreductase (POR), we chose to co-express each CYP3A isoform with POR to guarantee maximal CYP3A activity, for which TST and NIF were selected as marker substrates. TST is a prototypical CYP3A substrate in humans^53,54^, cattle, horse and pigs^52,55–63^. In particular, 6β-OH TST is the most common metabolite used to assess CYP3A-mediated activity. In cattle, TST 6β-hydroxylation has been confirmed to be specific for bCYP3A4/5-like activity by chemical and antibody-mediated inhibition studies^57^. The oxidation of NIF is another marker of human CYP3A metabolism^64,65^. However, the established human CYP3A4/5 substrate midazolam^54^ was not utilized because previous analytical investigations with bovine liver microsomes suggested poor specificity for CYP3A. Moreover, no significant correlation between midazolam metabolism and 6β-OH TST production was seen (Quintieri *et al*., manuscript in preparation).

Wild-type CYP3A28, CYP3A38 and CYP3A48 isozymes displayed a different behavior towards TST. CYP3A28 produces exclusively 6β-OH TST, whereas CYP3A38 yields both 6β-OH and 16β-OH TST derivatives. However, CYP3A48 is only marginally involved in TST metabolism, as only very low amounts of 2β-, 6β-, 12β- and 16β-hydroxylated metabolites were produced. Since no supporting literature was available, we sought confirmation from molecular modelling studies. Compared to the other two isoforms, CYP3A28 showed a much favorable docking score, orientation and distance to heme of the TST substrate, so that these factors could explain the higher enzymatic activity and specific production of 6β-OH TST. Predictions for CYP3A38 showed an intermediate docking score and a range of different orientations for TST, potentially explaining the lower total activity and formation of both 6β- and 16β-hydroxylated metabolites. Finally, for CYP3A48, we predicted a poor binding of TST, confirming the low efficiency of CYP3A48 isoform towards this substrate. On the other hand, NIF could be docked in the CYP3A48 substrate pocket. Overall, we can conclude that predictions of the molecular modeling correlated with the metabolic activity of the wild-type bCYP3A isozymes.

Five out of 13 variants showed an altered catalytic activity. The three CYP3A28 SNVs (CYP3A28_7 Gly197Ser, CYP3A28_10 Ala289Val, CYP3A28_11 Ile388Val) decreased the 6β-OH TST production by 50% as compared to the wild-type *CYP3A28*, while CYP3A38_11A Glu374Asp and CYP3A48_8B Val351Ile displayed three-fold increases in 16β-OH TST and oxidized nifedipine (NIF-OX) formation, respectively. Intriguingly, the frequency of CYP3A38_11A Glu374Asp variant was only 0.3% in the Piedmontese cohort. The rareness of this variant and the increase of its *in vitro* activity let us hypothesize that it might exert negative effects on bovine biology. Here, bulls homozygous for the CYP3A38_11A Glu374Asp variant were absent. Perhaps the MUT/MUT genotype is deleterious, while the heterozygous condition still guarantees presence of a functional CYP3A38 enzyme. Still, the absence of MUT/MUT animals might also be a consequence of limited sample numbers.

On a comparative basis, a wide number of missense SNVs throughout the entire human *CYP3A4* and *CYP3A5* coding sequences have been already described^2^. Overall, most of them showed a decreased CYP3A activity, while an increased activity is rarely reported. The cause for the increased catalytic activity is generally more difficult to explain and it might be substrate-dependent^2^. The missense mutations described for human *CYP3A4* and *CYP3A5* do not exactly match the positions of the variants in respective bovine orthologues *CYP3A28* and *CYP3A38*; moreover, the amino acid changes are not identical so as to permit a strict comparison. On the other hand, the C994G variant previously described in cattle by other authors^37^ perfectly matches with our CYP3A48_6B Val225Leu and with the reference SNV ***rs*110013281**. These authors supposed that the minor allele (G) in that position might cause a conformational change in the protein, which resulted in lowered CYP3A48 activity (genotype effect), toxin metabolism and/or milk production. Furthermore, because SNV C994G is not equally frequent among Angus, Brahman and reciprocal crosses, they hypothesized that there would be a heterosis advantage of using Angus × Brahman crossbred cows in forage systems that include toxic tall fescue^37^. However, they did not confirm these findings through evaluation of the functional activities of the WT and MUT gene products. Conversely, we characterized the same mutation thoroughly and showed that the CYP3A48_6B Val225Leu did not affect the protein function. According to the observed allele frequencies, we estimated that this variant was not in HWE in Piedmontese breed.

Most b*CYP3A* mutations led to only a modest (<50%) change in catalytic activities. This is in agreement with their great distance from the CYP3A active site^66^, the substrate recognition sites (SRSs^67^), the putative substrate access channels^68^ or from the arginine residues predicted to interact with POR^69^. Also, these mutations were predicted to be benign by the Polyphen-2 algorithm^70^.

The exceptions, showing the greatest changes in catalytic activity, were CYP3A38_11A Glu374Asp (258% increase in TST 16β-hydroxylation), CYP3A48_5 Asn180Thr and CYP3A48_8B Val351Ile (60% and 317% increase in NIF oxidation, respectively). Of these, CYP3A38_11A Glu374Asp was located near the putative substrate access channel and near the critical residues within the SRS-5 in CYP3A4^71^. It is plausible that a smaller amino acid (Asp) allows a better substrate access to the CYP active site. The Asp-to-Thr mutation in CYP3A48_5 Asn180Thr maps outside of SRS-4^72^ or other functional regions, and we do not have a clear explanation for the modest 60% increase in its activity. The Val-to-Ile mutation in CYP3A48_8B Val351Ile is located in helix L which harbors one of the conserved arginine residues for POR interaction, but it is difficult to predict why reaction rate would increase over 3-fold due to this quite conservative change. Finally, it should be underlined that CYP3As often display atypical kinetics and multiple substrate binding modes even for TST^73–75^. Therefore, the observed changes would require more detailed kinetic analysis before further mechanistic explanations can be suggested.

While V79 cells are a convenient platform to assess heterologously expressed CYP3A variants, naturally produced CYP3A isozymes in liver microsomes are more comparable to the *in vivo* condition^59^. Therefore, we evaluated TST hydroxylation in individual liver microsomes and matched the catalytic activities with the corresponding genotypes. Among the seven SNVs present as homozygotes, CYP3A28_7 Gly197Ser (***rs*384467435**) was the only example of a direct correspondence between *in vitro* and *ex vivo* results. Indeed, animals with this specific MUT/MUT genotype showed about a 50% lower pattern of TST 6β-hydroxylation when compared to the other genotype groups, in accordance with the results from V79 cells, although showing comparable amounts of CYP3A apoprotein. This suggests that the Gly197Ser mutation could be of interest for its potential consequences on xenobiotics metabolism *in vivo*. Noteworthy, this mutation was found at a similar frequency also in another population of Limousine cattle (Tolosi *et al*., submitted), confirming its occurrence and its potential relevance in different cattle breeds.

None of the other six SNVs present on the *CYP3A48* gene showed a change in the microsomal activity. This result could be rationalized by the fact that although CYP3A48 isoform is the most abundant in cattle liver^25^, it is only marginally involved in TST hydroxylation. The other hepatic CYP3A isoforms, less expressed but more efficient towards TST, are responsible for the bulk of TST hydroxylation, which minimizes any differences among the genetic subgroups. Unfortunately, no additional CYP3A isoform-specific substrates are actually available in cattle, as CYP3A-mediated microsomal metabolism has so far been assessed only with TST and midazolam [57; Quintieri *et al*., manuscript in preparation]. Further studies are definitively required to validate new probe substrates using bovine-specific antibodies and heterologous expression systems.

For the remaining four SNVs (CYP3A28_11 Ile388Val, CYP3A38_8 Val253Ile, CYP3A38_11B Phe376Leu and CYP3A48_7 Glu311Lys), heterozygotes did not show any effect on the microsomal activity; we may assume that none of these SNVs is deleterious, in accordance with predictive *in silico* analysis and modelling.

In conclusion, we identified the missense SNVs present in the b*CYP3A* gene cluster, and we assessed their functional impact on the oxidative metabolism of marker substrates by using both *in vitro* and *ex vivo* approaches. Among the 13 SNVs identified in Piedmontese bulls, only CYP3A28_7 Gly197Ser (***rs*384467435**) provided clear evidence of a functional impact on CYP3A metabolic capacity. The present work provides new information on cattle drug metabolism; moreover, it represents a framework for future pharmacogenetic investigations aiming to provide new knowledge on xenobiotic kinetics, therapeutic and adverse effects as well as consumer risk assessment.

## Methods

### Chemicals and reagents

Nifedipine (NIF), oxidized nifedipine (NIF-OX), TST, the internal deuterated standards TST and terfenadine, and β-nicotinamide adenine dinucleotide 2′-phosphate reduced tetrasodium salt hydrate (β-NADPH) were obtained from Sigma-Aldrich (St. Louis, MO, USA). 6β-hydroxytestosterone, (6β-OH TST), 16β-hydroxytestosterone (16β-OH TST) and 4-androsten-17α-OL-3-one (Epi-TST) were purchased from Steraloids (Newport, RI, USA).

For LC-MS/MS analyses, all reagents were of analytical grade: methanol (MeOH) was from Carlo Erba reagents (Milan, Italy) and formic acid (98%), dichloromethane and hexane from Sigma-Aldrich. Acetonitrile (HPLC grade), MeOH and dichloromethane for HPLC analyses were obtained from Mallinckrodt-Baker (Milan, Italy). All other reagents for microsomal incubations and HPLC analyses were obtained from Carlo Erba Reagents (Milan, Italy). Pure water was prepared from a Milli-Q water purification system (Millipore, Bedford, MA, USA).

Rabbit polyclonal antibody against human CYP3A43 (GTX117120) and human β-actin (ACTB, GTX109639) were purchased from Genetex (Irvine, CA, USA). Rabbit polyclonal antibody against human cytochrome P450 oxidoreductase (POR, H-300, sc-13984) was obtained from Santa Cruz Biotechnology (Dallas, TX, USA). Goat anti-rabbit horseradish peroxidase-labeled IgG (AP132P) and ChemiBlot^TM^ Molecular Weight Marker 2230-S were from Millipore (Burlington, MA, USA). PageRuler Plus Prestained Protein Ladder (code 26619) was from Thermo Scientific (Waltham, MA, USA).

### Animals

The study was carried out on 300 purebred Piedmontese young bulls (mean body weight ~550 kg at slaughter) that were the progeny of 25 sires. Each pedigree was composed of 3–22 bulls. Pedigree information was supplied by the Association of Piedmontese Cattle Breeders (ANABoRaPi, Carrù, Italy) and included animals with phenotypic records for carcass and meat quality and information of all their ancestors.

At the slaughterhouse, the same liver lobe was removed 30 minutes after stunning, and 5-gram samples were aseptically collected, immediately snap-frozen in liquid nitrogen and stored in the laboratory at −80 °C until genotyping and isolation of microsomal subcellular fractions.

### Genomic DNA isolation

Genomic DNA was isolated from frozen liver samples using Invisorb Spin tissue Mini Kit (Inviteck, Berlin, Germany) according to the manufacturer’s instructions. Genomic DNA concentration was measured using Qubit 2.0 Fluorometer and Qubit dsDNA BR Assay Kit (Life Technologies, Foster City, CA, USA). DNA quality was estimated by the 260/280 and 260/230 nm absorbance ratios (Nanodrop ND-1000 spectrophotometer; Nanodrop Technologies, Wilmington, DE, USA). For samples with either low DNA concentration or 260/280 and 260/230 nm absorbance ratios < 1.8, DNA extraction was repeated twice or three times so as to reach both amount and quality thresholds.

### Target enrichment and next-generation sequencing

Genomic DNAs isolated from 300 bulls was pooled into 16 samples. Pooling strategy was based on two main criteria: homogenous number of samples per pool (*i.e*., 18 or 19) and genetic homogeneity (*i.e*., each pool includes half-siblings fathered by maximally three different sires). To guarantee the detection of rare variants, all samples isolated from animals sharing the same sire were pooled together. A target enrichment approach was designed to selectively sequence b*CYP3A* genes. The b*CYP3A* gene cluster (chr25: 37,005,334–37,319,692, Bovine Genome release UMD3.1) including all exons, introns, intergenic and promoter regions was target-enriched using a custom Agilent SureSelect^XT^ Target Enrichment Kit (Agilent Technologies, Santa Clara, CA, USA) according to the manufacturer’s protocol. Quality control for sizing and concentration was performed using 2100 Bioanalyzer (Agilent Technologies, Santa Clara, CA, USA), and at least 500 ng of the amplified library was used for enrichment. For target enrichment, RNA oligonucleotides for solution hybrid selection (“baits”) were designed from Bovine Genome release UMD3.1 using SureDesign platform (Agilent Technologies, Santa Clara, CA, USA) adopting the following parameters: centered design strategy, bait length 120 nucleotides, bait tailing frequency 5X, allowed overlap into avoided regions for a maximum of 20 bp, antisense strand, and moderate masking. The SureSelect hybridization and capture was performed according to the manufacturer’s instructions. Libraries were sequenced using a 2 × 100 bp paired ends strategy on an Illumina HiSeq. 2500 sequencer (Illumina, San Diego, CA, USA) at IGA Technologies (Udine, Italy). To avoid potential biases related to divergent sequencing qualities, all the 16 barcoded libraries were loaded into two different lanes.

### Sequencing data analysis and variant detection

Briefly, the alignment of obtained DNA sequences to the reference bovine UMD 3.1 assembly was performed by BWA software^76^. Sequences that mapped to unique positions were retained and all the duplicate sequences (*i.e*. PCR artifacts) were removed. The Genome Analysis Tool Kit (GATK) local realignment tool^77^ was used to locally realign reads to minimize the number of mismatching bases across all the reads. This process served to transform regions with misalignments due to insertion/deletion (indels) into clean reads that contained a consensus indel suitable for standard variant discovery approaches^77^. Alignment and coverage statistics were produced by using Picard Toolkit (http://broadinstitute.github.io/picard/) and SAM tools^78^, respectively. The GATK tool “Unified-Genotyper” was used for variant calling, indels discovery and sample genotyping. Variant recalibration and filtering were performed by following the Broad Institute guidelines. Variants localized within b*CYP3A* cluster in the UMD3.1 assembly were finally selected and functionally annotated by ANNOVAR^79^.

### Melting curve genotyping

For validation of sequencing data and genotyping of the bovine cohort (n = 300), SNV-specific melting curve genotyping assays were set up using FRET HybProbe probes^80^. Specific assays for mutation detection were designed using LightCycler Probe Design Software (Roche, Basel, Switzerland) following recommendations of the producer. The probe and oligonucleotide primer sequences (Supplementary Table S2) were checked for specificity and thermodynamic characteristics using the standard tools such as NCBI BLAST, UCSC In silico PCR and OligoAnalyzer.

The optimization of Real time PCR assays was performed in a final volume of 10 µL containing up to 10 ng of template DNA, 5 µL of 2X LightCycler480 Probe Master Mix (Roche, Basel, Switzerland), 0.3–1.2 µM of each primer and 0.1–0.2 µM of each FRET probe (Roche, Basel, Switzerland). Assays were performed on a LightCycler480 real time PCR instrument (Roche, Basel, Switzerland) as follows: denaturation of DNA and Taq polymerase activation at 95 °C for 10 min, followed by 45 amplification cycles of 95 °C for 10 sec, 60 °C for 10 sec and 72 °C for 10 sec. The ramping temperature was set at 4.4 °C/sec for all steps except for the annealing, for which it was set at 2.2 °C/sec. After amplification, the melting curve analysis was performed. It consisted of four temperature steps: 95 °C for 1 min with a ramping rate of 4.4 °C/sec, 40 °C for 2 min at a ramping rate of 2.2 °C/sec and 95 °C for 0 sec with a ramping rate of 0.11 °C/sec, with continuous monitoring of the fluorescence on the 640 nm channel. A final step consisted of cooling at 2.2 °C/sec to 40 °C with a 30 sec hold.

For sample analysis, a negative control (water) and DNA samples belonging to individuals with a known genotype [homozygous wild-type (WT/WT) or mutant (MUT/MUT) or heterozygote (WT/MUT), if available in the population tested)] were added to each plate. Reference DNA samples, necessary for accurate genotyping, were selected after PCR end-point amplification, cloning, plasmid purification and confirmatory Sanger sequencing of at least ten colonies.

### Cloning of CYP3A28, CYP3A38, CYP3A48 and POR coding sequences

We selected liver samples (~100 mg) from four male bulls, each showing a WT/WT genotype for all three bCYP3A isoforms. Total RNA was isolated using TRIzol reagents (Life Technologies, Foster City, CA, USA) and then purified with RNeasy Mini kit (Qiagen, Hilden, Germany) following the manufacturers’ instructions. The concentration and quality of the RNA extracts were checked with a Nanodrop spectrophotometer. One µg of total RNA was reverse-transcribed using SuperScript® IV Reverse Transcriptase (Life Technologies, Foster City, CA, USA) and oligo d(T)_20_ primers. The complete coding sequence of *CYP3A28* (**NM_001099367**, **ENSBTAT00000063483.1**), *CYP3A38* (**NM_001075888**, **ENSBTAT00000007170.5**), *CYP3A48* (**NM_174531, ENSBTAT00000016177.5**) and *POR* (**ENSBTAT00000022718**) was then amplified using Phire Hot Start II DNA polymerase (Life Technologies, Foster City, CA, USA) and primer pairs, designed on the untranslated regions (UTRs) to guarantee an isoform-specific amplification (see oligonucleotides in Supplementary Table S3). The amplicon size was checked with 1.5% low-melting agarose gel electrophoresis. The specific PCR product was isolated and purified using High Pure PCR Product Purification kit (Roche, Basel, Switzerland).

To introduce restriction enzyme sites for cloning, nested PCR on the purified DNA was performed using Q5® High-Fidelity DNA Polymerase (New England BioLabs, Ipswich, MA, USA) and the primer pairs listed in Supplementary Table S4. Oligonucleotides were designed manually to contain appropriate restriction sites and to maintain the Kozak sequence upstream of the ATG start codon. The PCR products of *CYP3A28*, *CYP3A38* and *CYP3A48* were subcloned into the *Xho*I and *Mlu*I sites, while *POR* was inserted between *EcoR*I and *Sal*I sites of the pCI-neo expression vector. After plasmid purification with QIAprep Spin Miniprep kit (Qiagen, Hilden, Germany), orientation and sequence of the four genes was verified by Sanger sequencing.

### Site-directed mutagenesis

Site-directed mutagenesis of the 13 detected variants was performed by PCR, using *CYP3A28*, *CYP3A38* and *CYP3A48* wild-type clones as the template and using the In-Fusion HD Cloning kit (Clontech, Mountain View, CA, USA). Primers were designed following the manufacturer’s instructions and are reported in Supplementary Table S5. Mutated constructs were confirmed by Sanger sequencing.

### Transient transfection of bCYP3A clones in V79 cells

V79 Chinese hamster lung fibroblasts cells were obtained from European Collection of Authenticated Cell Cultures (ECACC, UK) and used for heterologous transfection of wild-type and mutant b*CYP3As* and *POR*. V79 cells (passages 15–25) were cultivated in high glucose Dulbecco’s Modified Eagle Medium (DMEM, Gibco 21969035), supplemented with 5% fetal bovine serum (FBS), 4 mM L-glutamine, 100 U/mL penicillin and 100 µg/mL streptomycin (all from Life Technologies, Foster City, CA, USA). Before transfection, cells were seeded in 6-well plates at a density of 90,000 cells/well in Opti-MEM supplemented with 5% FBS and cultured overnight to reach 50–70% confluence. About 24 h after seeding, cells were transfected for six hours with a mixture of DNA (1.2 µg of each b*CYP3A* construct + 120 ng of *POR* construct + 180 ng of pCI-neo empty vector per well) or with pCI-neo expression vector (1.5 µg/well) using FuGene HD Transfection Reagent (Promega, Madison, WI, USA) at a FuGene:DNA ratio of 3:1; then, the medium was replaced with complete DMEM. About 40 h post-transfection, the medium was changed again with 3 mL of complete DMEM without FBS, containing TST (25 µM) or NIF (5 µM). An incubation time of 3 or 1.5 h was used for TST and NIF, respectively. Subsequently, the medium was collected and stored at −20 °C for LC-MS/MS analysis. Four independent experiments were performed. Cells transfected with the empty pCI-neo vector and native untransfected cells were used as controls.

### Total protein isolation and immunoblotting

Total proteins were obtained from transfected V79 cell homogenates. After collection of the TST and NIF incubation medium, monolayers were washed with ice-cold PBS and scraped off with 100 µL of ice-cold lysis buffer (150 mM NaCl, 1% Triton X-100, 0.5% sodium deoxycholate, 0.1% SDS, 50 mM Tris pH 7.4). The cell suspension was agitated for 1 h at 4 °C, centrifuged 20 min at 12,000 rpm (4 °C) and the supernatant was collected. Protein content was measured using the BCA assay kit (Pierce, Life Technologies, Foster City, CA, USA).

Bovine liver microsomal proteins (10 µg) and proteins isolated from transfected and native V79 cells (60 µg) were resolved on SDS-polyacrylamide gels (4–12%) and transferred onto nitrocellulose membranes as previously reported^25^. After an overnight blocking in Tris-buffered saline buffer (TBS) containing 0.1% Tween-20 and 3% milk, membranes were first incubated for 2 h with rabbit anti-human CYP3A43 (dilution 1:500 for CYP3A48 and 1:1500 for CYP3A28 and CYP3A38), POR (1:2000) or ACTB (1:6000) polyclonal antibodies and then for 1.5 h with horseradish peroxidase-conjugated goat anti-rabbit IgG (1:6000 final dilution). The specific proteins were detected by using SuperSignal® West Pico chemiluminescence substrate (Pierce, Life Technologies, Foster City, CA, USA) according to manufacturer’s instructions.

### Catalytic activity of bCYP3As

After the incubation (*see Section 2.9*) of transfected and native cells with either TST (25 µM) or NIF (5 µM), the culture medium was spiked with the internal standard (IS, 1 μg/mL deuterated TST or terfenadine in MeOH) and then extracted with an equal volume (1 mL) of hexane:dichloromethane (1:1, v/v) by vortexing^81^. After centrifugation, the water phase was re-extracted. The combined organic phases were evaporated to dryness using a TurboVap evaporator (Zymarck, Hopkinton, MA). The residues were dissolved in 0.2 mL of 0.1% formic acid in 50% MeOH for LC-MS analysis.

The chromatographic separation was achieved using an Accela 600 HPLC pump with a CTC automatic injector (Thermo Fischer Scientific, San Jose, CA, USA) equipped with a Kinetex analytical column (100 × 2.1 mm, 1.7 µm; Phenomenex, Torrance, CA, USA). A gradient profile was applied using water with 0.1% formic acid (v/v) as solvent A and MeOH with 0.1% formic acid (v/v) as solvent B. The mobile phase composition (% A: % B) was as follows: 80:20 at 0 min, 40:60 at 2 min, 30:70 at 14 min, 5:95 at 15 min, held at 5:95 until 16.5 min, and then 80:20 from 17.50 min to 20 min to re-equilibrate the system. The samples were maintained at 4 °C, the injection volume was 10 µL, and the flow rate was set at 200 µL/min. The mass detection was achieved by a LTQ XL ion trap (Thermo Fischer Scientific, San Jose, CA, USA) equipped with a heated electrospray ionisation probe. The mass analyser was set in the full scan monitoring mode. The single standard solutions at 1 µg/mL were infused directly into the mass spectrometer via a syringe pump at a 20 µL/mL flow rate to find the fragmentation patterns and tuning parameters. Working solutions containing all the analytes (TST, 6β OH-TST, NIF, NIF-OX and terfenadine except for the deuterated TST IS) were used to spike blank samples to obtain the following concentrations: 0.69, 1.73, 3.46, 6.92, 17.3, 34.6, 69.2, 173 and 346 nM.

Calibration lines were constructed by plotting the ratio of the analyte area to the internal standard (IS) area versus the added concentrations (0.69–346 nM). A linear regression analysis was carried out to determine slope, intercept and coefficients of linear regression (r^2^). The LLOQ was 0.69 nM for all the analytes monitored.

The molecular weights, precursor ions, product ions, collision energies and retention times for all analytes are shown in Supplementary Table S6. The X-calibur software (version 2.1) was used for system control, data acquisition and analysis.

The optimal ratio of CYP3A to POR in terms of TST hydroxylation was investigated in pilot transfection studies. Different ratios between each single b*CYP3A* isoform and b*POR* construct (4:1, 6:1 and 10:1) were tested, with no significant differences observed in the metabolic rate. Therefore, the three wild-type isoforms and all mutants were analysed with the 10:1 ratio of CYP:POR. Pilot studies also define the TST and NIF final concentrations and proper incubation times in the further experiments.

The catalytic activity (nmoles min^−1^ mg^−1^ protein) in the medium was calculated by dividing the amount of metabolite present in culture medium by the incubation time and the total protein content. For each experiment, catalytic activities of mutant CYP3A28, CYP3A38 and CYP3A48 proteins were normalized to the activity of the corresponding wild-type protein.

### TST hydroxylation in bovine liver microsomal fractions

Microsomal subcellular fractions from liver samples (n = 300) were isolated by differential ultracentrifugation^82^ and stored at −80 °C until use. Protein concentration was determined using Qubit 2.0 Fluorometer and Qubit Protein Assay Kit (Life Technologies, Foster City, CA, USA).

The mixture containing TST 250 µM, bovine liver microsomal proteins (0.4 mg mL^−1^) and enzymatic cofactors was incubated for 10 min at 37 °C and extracted using 3 mL of ice-cold dichloromethane as reported^57^. The organic phase was transferred into new tubes and evaporated to dryness under an air stream at 50 °C using a TurboVap evaporator (Zymarck, Hopkinton, MA). The dried residues were dissolved in 100 µL of mobile phase, vortexed and analysed with a JASCO HPLC apparatus (Tokyo, Japan), equipped with a 2000 PLUS line pump, an auto-injector and an UV-VIS detector set at 247 nm. The separation of TST metabolites was achieved applying a constant isocratic mobile phase (30 min), consisting of ultrapure water (A; 60%), acetonitrile (B; 28%) and MeOH (C; 12%). Samples (10 μL) were injected into an X-Terra C18 column (150 mm × 3 mm, 5 μm; Waters, Milan, Italy) equipped with an XTerra MS Guard Column (3.0 × 20 mm, 5 μm; Waters). Standard and calibration curves were calculated within the range 0.5–25 µM, and a linear least square regression analysis was used to determine slope, intercept and coefficients of linear regression (r^2^). The limit of detection (LOD) and LOQ were 0.1 µM and 0.5 µM, respectively, for all the analytes monitored.

The quantitation of 6β-OH and 16β-OH TST metabolites was based on internal calibration with normalization of the peak area to the IS (epitestosterone, 20 µM). The enzymatic rate was expressed as nmoles of TST metabolite min^−1^ mg^−1^ of microsomal protein.

### Molecular modelling of TST and NIF docked into WT type and MUT CYP3As

The human CYP3A4 with co-crystallized progesterone substrate (PDB: 5A1R)^83^ was used as a model template. All bCYP models were constructed using Discovery Studio version 4.5. Resulting structures were processed with the Protein Preparation Wizard of the Schrödinger Maestro version 11.0.015 (Small-Molecule Drug Discovery Suite 2016–4, Schrödinger, LLC, New York, NY, 2016). In all models, the heme iron was bound to an oxygen atom. The correct assignment of OPLS2005 force field for the resulting iron-oxo species was done as follows: the heme iron was represented as Fe^3+^, and it was connected via zero-order bonds to six neighboring atoms: the four heme nitrogens, the conserved heme-binding cysteine sulfur, and the oxygen. Hydrogen bonds were assigned, and the prepared bCYP3A structures were minimized using the OPLS2005 force field and restrained minimization (heavy atom converging RMSD 0.30 Å). The TST and NIF 3D structures were generated using Maestro and prepared using LigPrep with the OPLS3 forcefield. These CYP substrates were ionized at pH 7.4, and desalted and tautomers were generated using Epik. Substrates were docked using the Maestro Induced Fit (Version 3.1, Glide Grid generation version 5.1), which docks them to the defined CYP protein with Glide; then, Prime Refinement processes a predefined number of best scoring poses and the CYP structure is relaxed. A sufficiently large grid, ~20 Å long in the middle of the substrate binding pocket and the heme porphyrin ring, was defined to ensure enough space for a large CYP substrate. Otherwise, default settings were used. All docking poses were visually inspected and ranked based on scoring poses that estimate the free energy change during substrate binding.

### Statistical analysis

Statistical analysis was performed using GraphPad Prism 5 (San Diego, California, USA). Unpaired T-test or ANOVA were used and followed, where appropriate, by the Tukey’s *post hoc* test. A *p*-value < 0.05 was considered as significant. The genotype frequencies of each variant were examined for deviations from HWE within the population using the χ^2^ test implemented in the online calculator (https://www.coursehero.com/file/8442059/Court-lab-HW-calculator/). A *p*-value < 0.05 indicated a deviation from HWE.

## Supplementary information

Supplementary Material
