# Supplementary material for: Functional impact of cytochrome P450 3A (CYP3A) missense variants in cattle
Source: Sci Rep. 2019 Dec 23;9:19672. doi: 10.1038/s41598-019-56271-8 (PMC6927969; doi:10.1038/s41598-019-56271-8)

Supplementary information

Functional impact of cytochrome P450 3A (CYP3A) missense variants in cattle

Mery Giantin[1], Minna Rahnasto-Rilla[2], Roberta Tolosi[1], Lorena Lucatello[1], Marianna Pauletto[1], Giorgia Guerra[1], Francesca Pezzato[1], Rosa M. Lopparelli[1], Roberta Merlanti[1], Paolo Carnier[1], Francesca Capolongo[1], Paavo Honkakoski[2,3], and Mauro Dacasto[1,*]

[1]Department of Comparative Biomedicine and Food Science, Division of Pharmacology and Toxicology, University of Padua, Italy

[2]Faculty of Health Sciences, School of Pharmacy, University of Eastern Finland and Biocenter Kuopio, Kuopio, Finland

[3]Division of Pharmacotherapy and Experimental Therapeutics, Eshelman School of Pharmacy, University of North Carolina at Chapel Hill, Chapel Hill, NC 27599, U.S.A.

*mauro.dacasto@unipd.it

Supplementary Tables

Supplementary Table S1. TST hydroxylation in Piedmontese cattle liver microsomes.

SNV ID	GENOTYPE	6β-OH TST (nmol/min/mg prot)	16β-OH TST (nmol/min/mg prot)
CYP3A28_7 Gly197Ser	WT/WT (n = 236)	0.258 ± 0.011[bb]	0.185 ± 0.015
	WT/MUT (n = 53)	0.223 ± 0.021[c]	0.149 ± 0.022
	MUT/MUT (n = 10)	0.103 ± 0.013	0.059 ± 0.018
CYP3A28_11 Ile388Val	WT/WT (n = 258)	0.250 ± 0.017	0.155 ± 0.013
	WT/MUT (n= 39)	0.240 ± 0.023	0.147 ± 0.025
CYP3A38_8 Val253Ile	WT/WT (n = 264)	0.249 ± 0.017	0.156 ± 0.012
	WT/MUT (n = 36)	0.235 ± 0.024	0.134 ± 0.024
CYP3A38_11B Phe376Leu	WT/WT (n = 293)	0.246 ± 0.016	0.152 ± 0.012
	WT/MUT (n = 7)	0.295 ± 0.085	0.194 ± 0.091
CYP3A48_5 Asn180Thr	WT/WT (n = 176)	0.232 ± 0.013	0.149 ± 0.016
	WT/MUT (n = 121)	0.270 ± 0.033	0.162 ± 0.016
	MUT/MUT (n = 3)	0.204 ± 0.103	0.047 ± 0.017
CYP3A48_6A His220Asp	WT/WT (n = 253)	0.255 ± 0.011	0.178 ± 0.016
	WT/MUT (n = 35)	0.369 ± 0.108	0.193 ± 0.036
	MUT/MUT (n = 12)	0.211 ± 0.046	0.242 ± 0.053
CYP3A48_6B Val225Leu	WT/WT (n =100)	0.273 ± 0.020	0.184 ± 0.023
	WT/MUT (n = 119)	0.257 ± 0.034	0.147 ± 0.019
	MUT/MUT (n = 81)	0.223 ± 0.015	0.150 ± 0.017
CYP3A48_7 Glu311Lys	WT/WT (n = 293)	0.250 ± 0.016	0.155 ± 0.012
	WT/MUT (n =7)	0.138 ± 0.028	0.081 ± 0.029
CYP3A48_8A Asn316Lys	WT/WT (n = 174)	0.222 ± 0.013	0.153 ± 0.017
	WT/MUT (n = 113)	0.285 ± 0.035	0.153 ± 0.016
	MUT/MUT (n = 5)	0.314 ± 0.040	0.184 ± 0.040
CYP3A48_8B Val351Ile	WT/WT (n = 212)	0.231 ± 0.012	0.155 ± 0.014
	WT/MUT (n = 76)	0.295 ± 0.051	0.150 ± 0.020
	MUT/MUT (n = 11)	0.225 ± 0.020	0.143 ± 0.033
CYP3A48_9 Gly391Asp	WT/WT (n = 163)	0.247 ± 0.015	0.179 ± 0.017

	WT/MUT (n = 115)	0.195 ± 0.013	0.160 ± 0.020
	MUT/MUT (n = 22)	0.212 ± 0.036	0.160 ± 0.028

Data are expressed as arithmetic mean ± SEM. Statistical Analysis: ANOVA + Tukey's post test (3 groups) or Unpaired T-test (2 groups)
a: WT/WT vs WT/MUT; b: WT/WT vs MUT/MUT; c: WT/MUT vs MUT/MUT

Supplementary Table S2. Oligonucleotides and FRET probes for genotyping assays

ASSAY	Primer and probe sequence (5’-3’)	Length (bp)	GC %	Tm (°C)
CYP3A28_7 Gly197Ser	F: CTCAGCATCTTTGGAGCC	18	55.6	59.7
	R: GATACTTACCTACTGCGAGAAGAAA	25	40.0	60.0
	Sensor probe: LC640-TGCCGAGCGAATCAATATTCACTCC-PH	25	48.0	64.2
	Anchor probe: CTTCTTGACATTCTCCACAAAGGGATCTTGTGG-FL	33	45.5	68.8
CYP3A28_10 Ala289Val	F: ACTGGAATGGCTCATCATTTAAG	23	39.1	60.8
	R: GCCAAAATATACAGAAGGAAGGA	23	39.1	59.0
	Sensor probe: LC640-GCTATGAGTTCTTGGTCAGAGAGAGCTA-PH	28	46.4	60.6
	Anchor probe: GCTAGTGGTCTCATAGCCAGCAAAAATAAAGATAATACTCTG-FL	42	38.1	67.9
CYP3A28_11 Ile388Val	F: CGTGTAAAATGTCTATAAATGCTAACG	27	33.3	60.2
	R: TACCCTTTCAGGACGGAAC	19	52.6	60.5
	Sensor probe: GGGTGTCCATTCCCAAAGGGACAA-FL	24	54.2	66.6
	Anchor probe: LC640-GTGATGGTGCCAATCTCCGTGCTGC-PH	25	60.0	71.1
CYP3A38_8 Val253Ile	F: TCTTCAGTACTCTTTCCATTCCT	23	39.1	59.6
	R: GTGAACACCCTCATGTAACC	20	50.0	60.0
	Sensor probe: GGCGACTTTCTTTTATCTTTTTTACGGATGTT-FL	32	34.4	65.1
	Anchor probe: LC640-CAAAAAATTCACAGCACTTTTTGGAAATATGCTGA-PH	35	31.4	68.0
CYP3A38_11A Glu374Asp	F: AATCTATTTCTTTCTTCCCAGGC	23	39.1	60.1
CYP3A38_11B Phe376Leu	R: GCTGTGGGTCTCTGTGA	17	58.8	59.9
	Sensor probe: CCTCTCAAGTCTAACAGCAATAGGAAACATTCTGA-FL	35	40.0	65.6
	Anchor probe: LC640-GTCTCATTCACCACCATGTCAAGATACTCCATCTG-PH	35	45.7	68.5

CYP3A48_5 Asn180Thr	F: AGGAAAGAAGTTAAATATACACTCCTG	27	33.3	60.1
	R: TGAGAACCGCACCCTAC	17	58.8	60.4
	Sensor probe: ATGGTTGTCAATTTCTTTGGAATTCTGAGAATTA-FL	34	29.4	63.9
	Anchor probe: LC640-CATTAGCTGAAGTAAATCCACTCGATTCTGGAAAC-PH	35	40.0	66.7
CYP3A48_6A His220Asp<br>CYP3A48_6B Val225Leu	F: TCGTGGCCCAAAGTACTAT	19	47.4	59.6
	R: GGCTTCAACTTCTCTTCTCT	20	45.0	58.9
	Sensor probe: CCCTCATGTCCAGCAGAAGGT-FL	21	57.1	60.1
	Anchor probe: LC640-AGGAGGAAATTGATGCAACTTTCCCTAATAAGGTG-PH	35	40.0	67.5
CYP3A48_7 Glu311Lys	F: CCAAAGGGACAACCGTG	17	58.8	60.4
	R: TCACCCGTTTGACACCTATAAT	22	40.9	60.3
	Sensor probe: LC640-CTTTCGGGACGGAACTCCTCG-PH	21	61.9	64.0
	Anchor probe: GGCGTCTCTTCTCCAGGGTCTTGT-FL	24	58.3	64.7
CYP3A48_8A Asn316Lys	F: GAAATCTCCTTATGCTACAGGT	22	40.9	59.4
	R: GGTCCAGTTCCAAAAGGC	18	55.6	60.3
	Sensor probe: CTGTCCTTGTTCTTCTTACTGAACCTGG-FL	28	46.4	62.0
	Anchor probe: LC640-CCACAACAGTAGAGGAAACAAGTGAATGAAACAGA-PH	35	40.0	67.2
CYP3A48_8B Val351Ile	F: CGAAACTGCCTTGGCAT	17	52.9	60.7
	R: CATCAGTTAAAAAACAACTCCCAAT	25	32.0	59.5
	Sensor probe: LC640-TGACAAGAGCAAGTTTTATGTTCATGATGGCA-PH	32	37.5	66.9
	Anchor probe: CTTTACAAGGTTTGAAGGAGAAGTTCTGCAGGAT-FL	34	41.2	67.3
CYP3A48_9 Gly391Asp	F: TTATACACTCAAGGACTCACGC	22	45.5	60.1
	R: ACAGTAGGTGAAGCCCAT	18	50.0	60.0
	Sensor probe: LC640- CCAGGCCTCTGGGCACA-PH	17	70.6	61.6
	Anchor probe: TAGAGAAAGTCAGGCTCCACCTGTGG-FL	26	53.8	64.0

F: forward primer; R: reverse primer

Supplementary Table S3. Primer pairs used for the amplification of the complete coding sequence of b*CYP3As* and *POR*

**GENE**	**Primer**	**Primer sequence 5'-3'**	**Length (bp)**	**GC%**	**Tm (°C)**
CYP3A28	CYP3A28_FS_F	AGAGGCAGGCAGAGGGCA	18	66.7	70
	CYP3A28_FS_R	CACAAAGTAAATCAAGCCCCCTGAAATTC	29	41.4	67
CYP3A38	CYP3A38_FS_F	GAGAGGCAGGCAGAGAACAT	20	59.5	65
	CYP3A38_FS_R	AGTAAATCAAGTCCCCTGAAACTG	24	58.7	63
CYP3A48	CYP3A48_FS_F	GAGTGTGAAAGGAAGCAAGTGA	22	45.5	63
	CYP3A48_FS_R	AAAGTAGACCACGTCTCTGGTG	22	50.0	64
POR	POR_FS_F	ACGCCTGTGATTCCTGATGA	20	50.0	64
	POR_FS_R	GTGAGGCGCGAGATTACATG	20	55.0	64

F: forward primer; R: reverse primer

* estimated with NEB calculator tool (http://tmcalculator.neb.com/#!/)

Supplementary Table S4. Primer pairs used for cloning b*CYP3As* and *POR*

GENE	Primer	Primer sequence 5'-3'	Restriction enzyme	Length (bp)	GC%*	Tm (°C)*
CYP3A28	CYP3A28_RE_F	CGC**CtcGAg**AAGTGGCC*ATG*GAGTTAATCCTGAG	XhoI	34	48	69
	CYP3A28_RE_R	CAGGTCTT**acGcGt**AAG*TCA*GGCTCCACTTATGGTTC	MluI	36	48	67
CYP3A38	CYP3A38_RE_F	GC**CtcGAg**AAGTGGCC*ATG*GAGCTAATCCCA	XhoI	31	52	71
	CYP3A38_RE_R	CAGGTCCTT**AcGcGt**AG*TCA*AGCTCCACTTATGGTCT	MluI	37	45	65
CYP3A48	CYP3A48_RE_F	ATCA**CtcgAG**AAAGGGGCC*ATG*GAACTG	XhoI	28	55	68
	CYP3A48_RE_R	CGAAGT**aCgcgT**CC*TTA*GAGAAAGTCAGGCTC	MluI	32	48	63
POR	POR_RE_F	GCCTG**gaATTC**CTG*ATG*AACATGGCAGACT	EcoRI	30	43	66
	POR_RE_R	CAGTGG**GtCGaC***CTA*GCTCCATACGTC	SalI	27	56	58

* estimated with NEB calculator tool (http://tmcalculator.neb.com/#!/), considering only the underlined sequence (perfect alignment)

F: forward primer; R: reverse primer

Bolded: restriction enzyme site

Italics, light grey: the first codon (ATG) or the stop codon (TCA, TTA, CTA)

Lowercase letters: modifications introduced for creating restriction enzyme sites

Supplementary Table S5. Primer pairs for the site-directed mutagenesis of b*CYP3As*

ASSAY	Primer sequence (5'-3')	Length (bp)	GC %*	Tm (°C)*
CYP3A28_7	F: TCaGCAACCCACAAGATCCCTTTGTGGAGAATGTCAAGAAG	41	40.0	56.4
Gly197Ser	R: TTGTGGGTTGCtGAGCGAATCAATATTCACTCCAAATGATGTGC	44	36.0	53.9
CYP3A28_10	F: ATAAAGtTCTCTCTGACCAAGAACTCATAGCCCAGAGTATTATC	44	43.3	58.0
Ala289Val	R: AGAGAGAaCTTTATGATTGTCTGTTTCTTTGGAATTCTGGGAGT	44	40.0	58.9
CYP3A28_11	F: GTCCgTTCCCAAAGGGACAACAGTGATGGTGCCAATCTC	39	50.0	58.2
Ile388Val	R: CCTTTGGGAAcGGACACCCCATGGATTTCCACATCCTT	38	47.8	58.7
CYP3A38_8	F: CaTAAAAAAGATAAAAGAAAGTCGCCTCAAAGATACTCAAAAGC	44	41.4	58.9
Val253Ile	R: TTTATCTTTTTTAtGGATGTTGTCAAAAAATTCACAGCACTTTTTGG	47	34.4	58.1
CYP3A38_11A	F: AtAGGTTCTGTAAGAAGGATGTGGAAATCCATGGGGTGT	39	50.0	60.5
Glu374Asp	R: TCTTACAGAACCTaTCAAGTCTAACAGCAATAGGAAACATTCTGAGAG	48	39.4	58.6
CYP3A38_11B	F: AGGcTCTGTAAGAAGGATGTGGAAATCCATGGGGTGTC	38	52.2	58.0
Phe376Leu	R: CTTCTTACAGAgCCTCTCAAGTCTAACAGCAATAGGAAACATTCTGAGA	49	38.2	59.3
CYP3A48_5	F: AAAGAAATTGACAcCCATAAAGCTCTGTCTGACATAGAACTCGTG	45	43.3	59.3
Asn180Thr	R: gGTGTCAATTTCTTTGGAATTCTGAGAATTAATCATTAGCTGAAGTAAATCCA	53	33.3	58.8
CYP3A48_6A	F: TgATGTCCAGCAGAAGGTGCAGGAGGAAATTGATGCAAC	39	50.0	58.9
His220Asp	R: TTCTGCTGGACATcAGGGTGAGTGGTCAATTCATATATAATGAAGGA	47	37.5	57.9
CYP3A48_6B	F: CCAGCAGAAGcTGCAGGAGGAAATTGATGCAACTTTCCCTAATAAGG	47	40.6	59.2
Val225Leu	R: TGCAgCTTCTGCTGGACATGAGGGTGAGTGGTCAATTCATAT	42	40.7	57.9
CYP3A48_7	F: CCCaAAAGGTTCAGTAAGAATAACAAGGACAGCATAAATCCTTACGTC	48	37.5	58.3
Glu311Lys	R: TACTGAACCTTTtGGGACGGAACTCCTCGGGCTC	34	66.7	59.6
CYP3A48_8A	F: CAGTAAGAAgAACAAGGACAGCATAAATCCTTACGTCTACCTG	43	46.4	58.5
Asn316Lys	R: TTGTTcTTCTTACTGAACCTTTCGGGACGGAACTCC	36	57.1	59.4
	F: AAAACTTGCTCTTaTCAGAATCCTGCAGAACTTCTCCTTCAAACC	45	46.7	60.7

CYP3A48_8B Val351Ile	R: AtAAGAGCAAGTTTTATGTTCATGATGGCAAACCTCATGCC	41	46.2	60.1
CYP3A48_9 Gly391Asp	F: CAGAGaCCTGGGACCACAGGTGGAGCCTGACTTTCTC	37	54.5	58.8
	R: GGTCCCAGGtCTCTGGGCACAACCTTCAGAATAACAGGTTG	41	46.2	58.2

* calculated for the sequence in 3'-termini (underlined sequence excluded)

F: forward primer; R: reverse primer

Lowercase letters: genetic variant introduced with site-directed mutagenesis

Underlined sequence: ~15-bp homologous overlap at the 5'-termini of both primer F and R

Supplementary Table S6. Analytical standards and parameters used for LC-MS/MS data acquisition.

**Standard**	**Molecular weight**	**Precursor Ions**	**Collision Energies**	**Product Ions (MS2)**	**Retention time (min)**
TST	288.42	289	25	271, 253, 109, 97	9.9
6β-OH TST	304.42	305	30	287, 269, 251	6.0
16β-OH TST	304.42	305	30	287, 269, 251, 109, 97	6.9
DEUTERATED TST (IS)	291.44	292	35	274, 256, 109, 97	9.9
NIF	346.33	347	32	315, 254	7.2
NIF-OX	344.32	345	38	284, 269, 256, 237	6.2
TERFENADINE (IS)	471.67	472	33	454, 436	8.7

TST = testosterone; 6β-OH TST = 6β-hydroxytestosterone; 16β-OH TST = 16β-hydroxy-testosterone; IS = internal standard; NIF = nifedipine; NIF-OX = oxidized nifedipine

Supplementary Figures

Supplementary Figure S1: LC-MS/MS chromatograms obtained after the analysis of the cell medium spiked with the analytical standards, 6β- and 16β-OH TST (a) or with 25 µM TST and incubated with native V79 cells (b), CYP3A28- (c), CYP3A38- (d) and CYP3A48-transfected cells (e) for 3 hours. The metabolite of interest is shown by grey shading. The presence of 6β-OH TST metabolite (arrow) in non-transfected cells is due to an impurity of the analytical standard. 6β-OH TST = 6β-hydroxytestosterone; 16β-OH TST = 16β-hydroxytestosterone. RT = retention time. AA/MA = area amount.

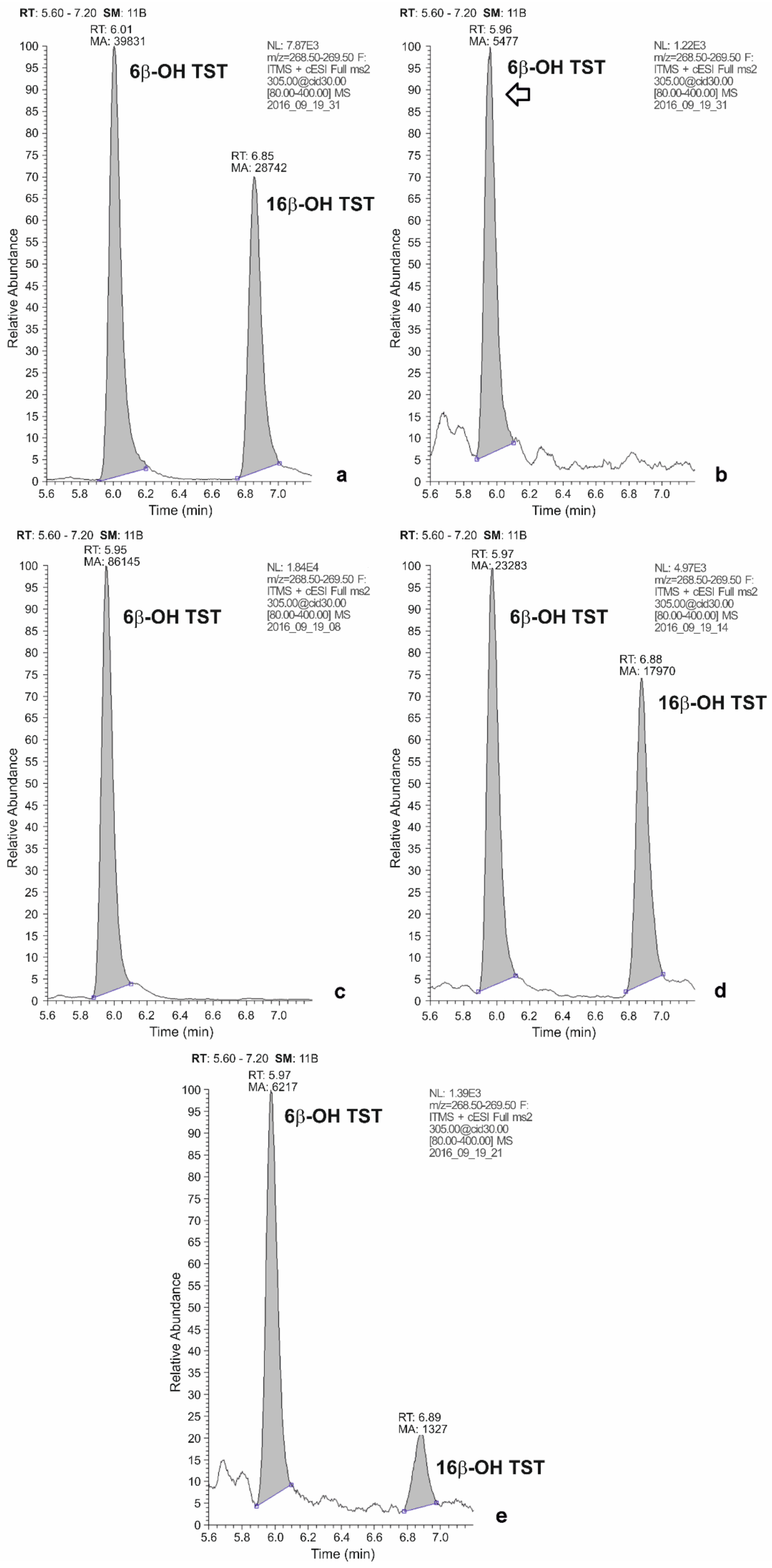

RT: 5.60 - 7.20 SM: 11B
RT: 6.01
MA: 39831
6β-OH TST
NL: 7.87E3
m/z=268.50-269.50 F:
ITMS + cESI Full ms2
305.00@cid30.00
[80.00-400.00] MS
2016_09_19_31
RT: 6.85
MA: 28742
16β-OH TST
Relative Abundance
Time (min)
a
RT: 5.60 - 7.20 SM: 11B
RT: 5.96
MA: 5477
6β-OH TST
NL: 1.22E3
m/z=268.50-269.50 F:
ITMS + cESI Full ms2
305.00@cid30.00
[80.00-400.00] MS
2016_09_19_31
Relative Abundance
Time (min)
b
RT: 5.60 - 7.20 SM: 11B
RT: 5.95
MA: 86145
6β-OH TST
NL: 1.84E4
m/z=268.50-269.50 F:
ITMS + cESI Full ms2
305.00@cid30.00
[80.00-400.00] MS
2016_09_19_08
Relative Abundance
Time (min)
c
RT: 5.60 - 7.20 SM: 11B
RT: 5.97
MA: 23283
6β-OH TST
NL: 4.97E3
m/z=268.50-269.50 F:
ITMS + cESI Full ms2
305.00@cid30.00
[80.00-400.00] MS
2016_09_19_14
RT: 6.88
MA: 17970
16β-OH TST
Relative Abundance
Time (min)
d
RT: 5.60 - 7.20 SM: 11B
RT: 5.97
MA: 6217
6β-OH TST
NL: 1.39E3
m/z=268.50-269.50 F:
ITMS + cESI Full ms2
305.00@cid30.00
[80.00-400.00] MS
2016_09_19_21
RT: 6.89
MA: 1327
16β-OH TST
Relative Abundance
Time (min)
e

Supplementary Figure S2: LC-MS/MS chromatograms obtained after the analysis of the cell medium spiked with the analytical standard, NIF-OX (a) or with 5 µM NIF and incubated with native V79 cells (b) and CYP3A48-transfected cells (c) for 1.5 hours. The metabolite of interest is shown by grey shading. The two white peaks on the right are indicative of further NIF metabolites or products of NIF degradation. NIF-OX = oxidized nifedipine. RT = retention time. AA/MA = area amount.

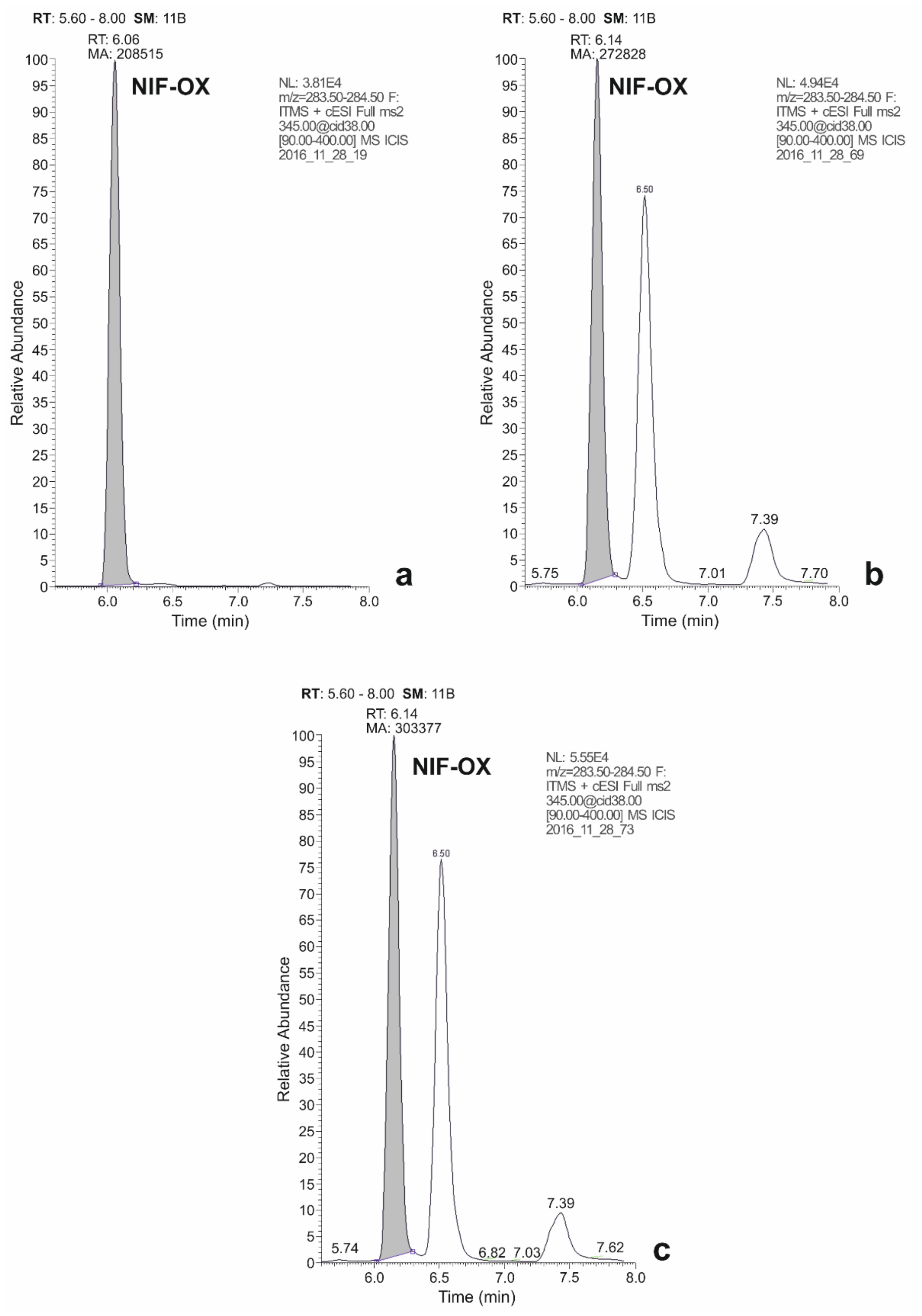

Supplementary Figure S3: Catalytic activity of V79 cells expressing wild-type and mutant CYP3A38. After incubation of transfected V79 cells with 25 µM 6β OH-TST for 3 hours, the medium was subjected to LC-MS/MS analysis as reported in the Material and Methods. The catalytic activity of wild-type and mutant CYP3A38 proteins was calculated dividing the amount of metabolite formed by the incubation time and the total protein content (nmoles min^{-1} mg^{-1} protein). Variant CYP3A specific activities were normalized to that of the wild-type CYP3A isoform (set at 1.00). Four independent experiments were performed. Unpaired T-test was used for statistical analysis. AU: Arbitrary Units

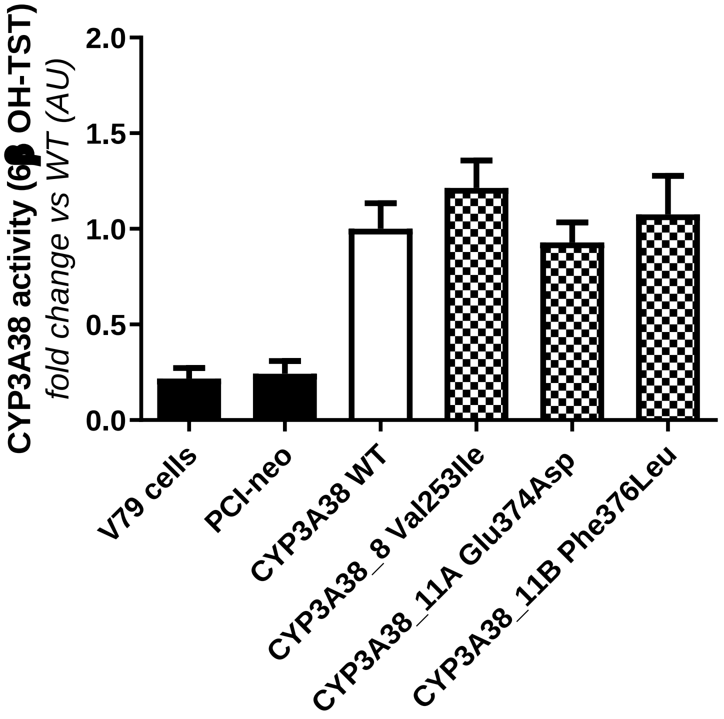

Supplementary Figure S4: CYP3A immunoblotting of liver microsomes from Piedmontese cattle genotyped for CYP3A28_7 Gly197Ser variant. A. Bovine liver microsomal fractions from 10 WT/WT (*w/w*), 10 WT/MUT (*w/m*) and 10 MUT/MUT (*m/m*) were isolated according to Pegolo et al. (2010). Proteins (30 µg) were subjected to immunoblotting analysis following the protocol reported in the Material and Methods section. Membranes were probed with an anti-human CYP3A43 and ACTB polyclonal antibodies raised in rabbit. A control sample, consisting of microsomal proteins obtained from the liver of untreated rats (Rat), was run to allow the densitometric analysis as well as the normalization of results, particularly for any developing and fixing discrepancies among different blots. Microsomes from dexamethasone treated rats (DEX) were also used as positive control. B. Scatter dot plot of densitometric values. Data are expressed in arbitrary units as mean ± SEM. Statistical analysis: ANOVA + Tukey's post-test.

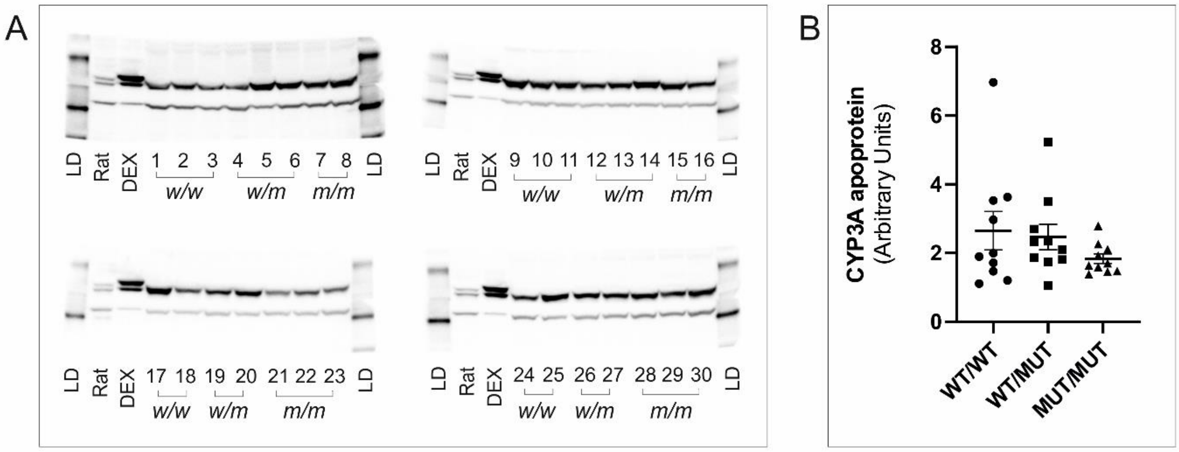

Supplementary Figure S5: close-up view of the alternative docking pose of testosterone in the CYP3A38 pocket. Here, the steroid D ring is projected towards the heme enabling hydroxylation at the C16 atom. Heme is depicted in *blue*, testosterone in *magenta* and CYP backbone in *grey* ribbon.

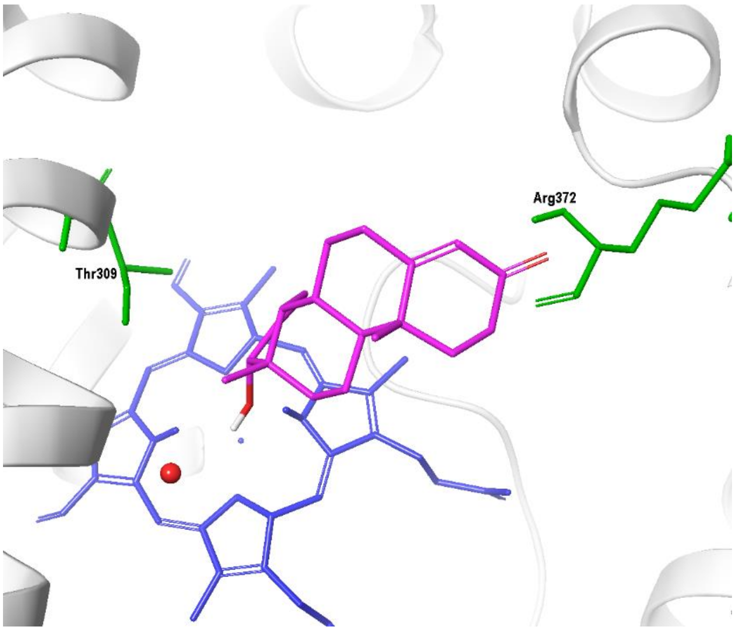

Supplementary Figure S6: docking poses of testosterone and residues within 3 Å distance in the substrate pocket of (A) CYP3A28, (B) CYP3A28.7, (C) CYP3A28.10 and (D) CYP3A28.11. Purple arrows indicate hydrogen bonding. Colors indicate hydrophobic (*green*), polar (*light blue*), cationic (*dark blue*) and anionic (*red*) residues or the heme group (*grey*).

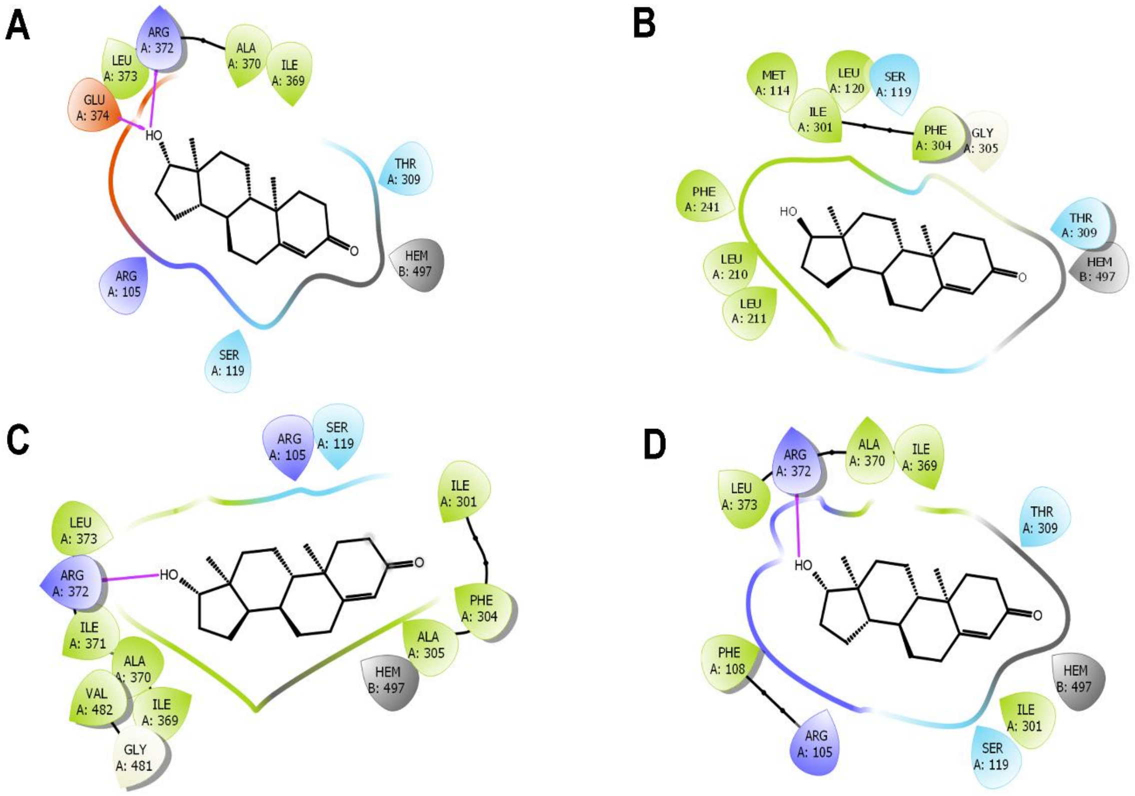

Supplementary Figure S7: docking poses of testosterone and residues within 3 Å distance in the substrate pocket of (A) CYP3A38, (B) CYP3A38.8, (C) CYP3A38.11A and (D) CYP3A38.11B. Purple arrows indicate hydrogen bonding. Colors indicate hydrophobic (*green*), polar (*light blue*), cationic (*dark blue*) and anionic (*red*) residues or the heme group (*grey*).

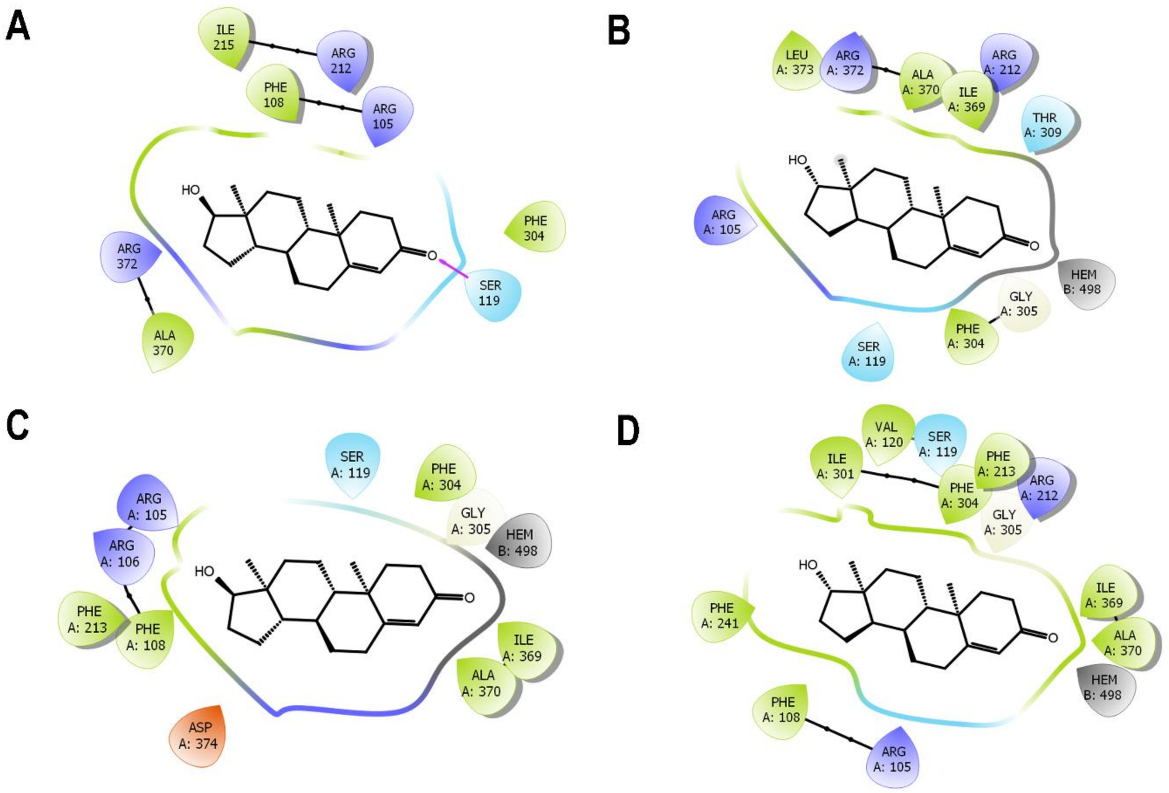

Supplementary Figure S8: the location of the binding pocket (*blue area*) and SNPs in the (A) CYP3A28, (B) CYP3A38 and (C) CYP3A48. Testosterone (*magenta*) and the heme porphyrin ring (*blue*) are also indicated. The CYP backbone is displayed by *grey* ribbon.

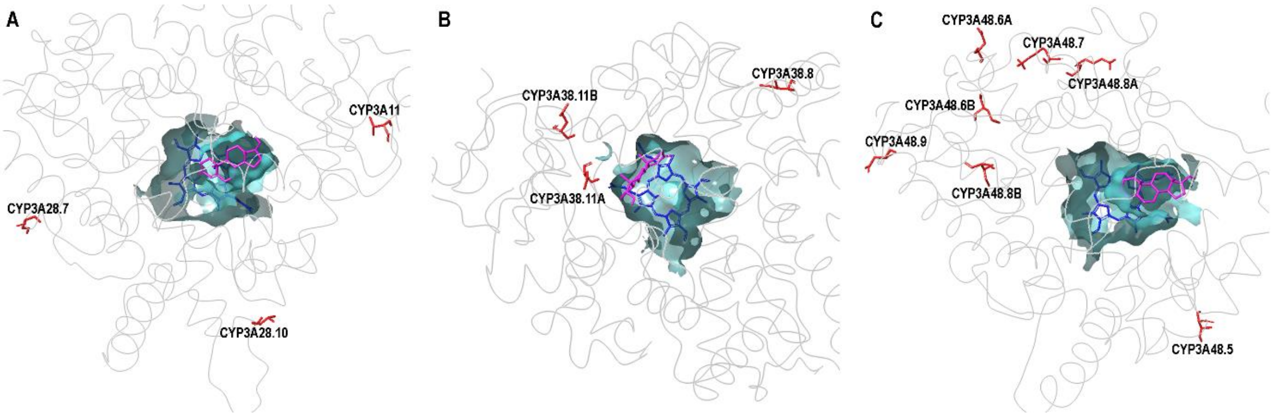

Supplementary Figure S9: docking poses of NIF and residues within 3 Å distance in the substrate pocket of (A) wild type CYP3A48, (B) CYP3A48_5 Asn180Thr, (C) CYP3A48_6A His220Asp, (D) CYP3A48_6B Val225Leu, (E) CYP3A48_7 Glu311Lys, (F) CYP3A48_8A Asn316Lys, (G) CYP3A48_8B Val351Ile, (H) CYP3A48_9 Gly391Asp. *Purple* arrows indicate hydrogen bonding, *red* lines π-cation, *green* lines π-π- stacking and *red/blue* gradient lines salt bridges.

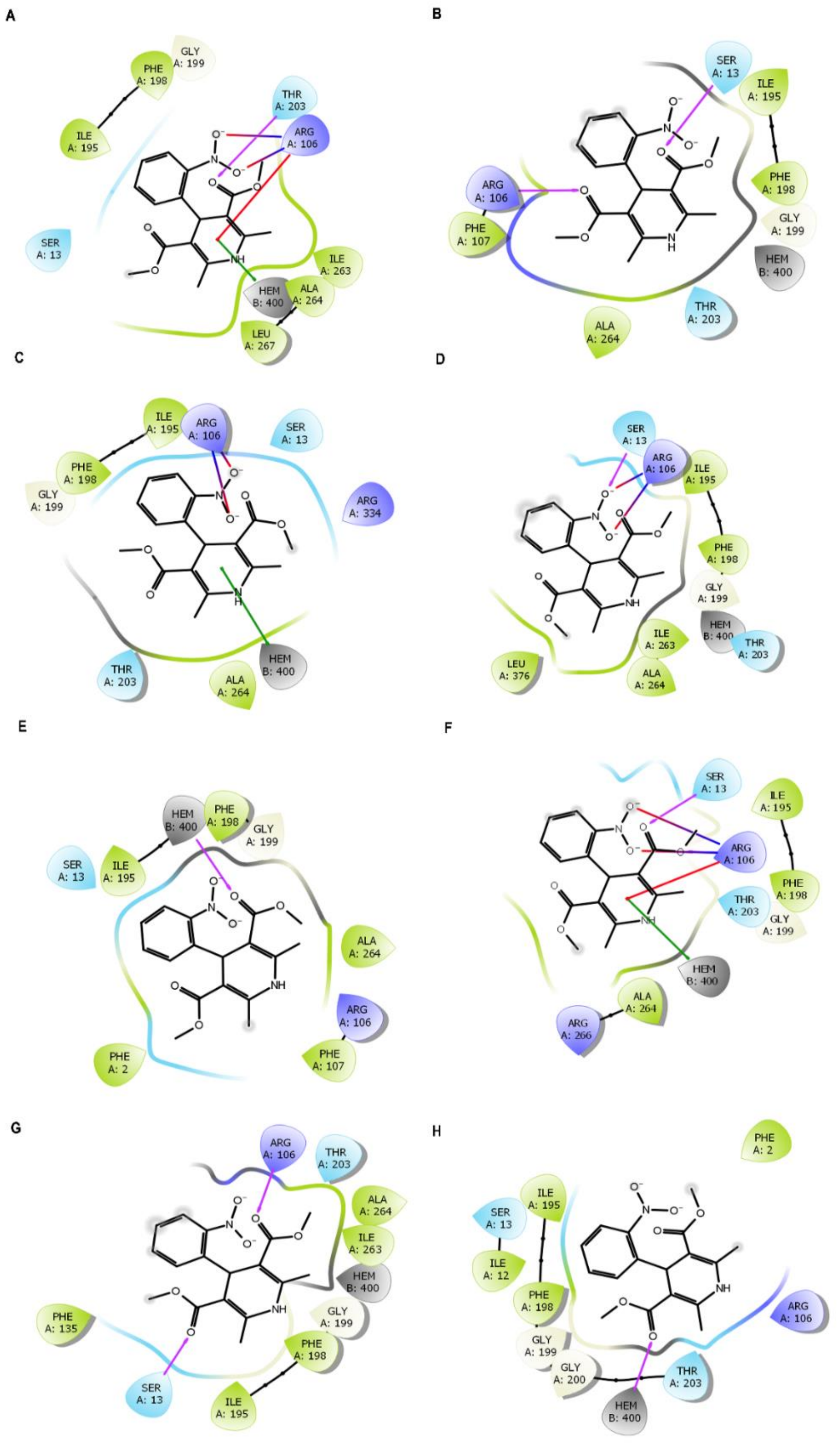

Supplement: Supplementary file 1 — Supplementary Material [file 41598_2019_56271_MOESM1_ESM.pdf]
